# Short‐Course Subcutaneous Immunotherapy With Mannan‐Conjugated Birch Pollen Allergoids: Consistent Clinical and Immunological Improvements

**DOI:** 10.1002/clt2.70209

**Published:** 2026-10-04

**Authors:** Esther Raskopf, Sofia Passamera, Ludger Klimek, Oliver Pfaar, Christian Neuhof, Anna Rybachuk, Nadine Katzke, Hacer Sahin, Silke Allekotte, Ralph Mösges, Laura Day, José Luis Subiza, Miguel Casanovas, Mandy Cuevas, Sandra del Pozo

**Affiliations:** ^1^ ClinCompetence Cologne GmbH Cologne Germany; ^2^ Faculty of Medicine Institute of Medical Statistics and Computational Biology University of Cologne Cologne Germany; ^3^ Center for Rhinology & Allergology Wiesbaden Germany; ^4^ Department of Otorhinolaryngology, Head and Neck Surgery Section of Rhinology and Allergy University Hospital Marburg Philipps‐Universität Marburg Marburg Germany; ^5^ Inmunotek S.L. Alcalá de Henares Madrid Spain; ^6^ Department of Otorhinolaryngology, Head and Neck Surgery Faculty of Medicine (and University, Hospital) Carl Gustav Carus TUD Dresden University of Technology Dresden Germany

**Keywords:** allergic rhinoconjunctivitis, birch pollen, combined symptom and medication score, mannan‐conjugated allergoid, subcutaneous immunotherapy

## Abstract

**Background:**

The mannan‐conjugated birch pollen polymerised allergoid EP‐088_T502 has previously been shown to reduce allergic symptoms and anti‐allergic medication use during the birch pollen season. This confirmatory phase III trial aimed to evaluate the efficacy, safety, tolerability, and immunologic effects of EP‐088_T502 administered as eight subcutaneous injections over six pre‐seasonal treatment visits.

**Methods:**

In this double‐blind, placebo‐controlled (DBPC) trial, 278 participants with birch pollen–induced allergic rhinoconjunctivitis (ARC) received either placebo or a cumulative dose of 28,000 mannan therapeutic units (mTU) of EP‐088_T502. The primary efficacy endpoint was the combined symptom and medication score (CSMS) during the peak birch pollen season. Safety, tolerability, health‐related quality of life (QoL), and immunologic parameters were also assessed.

**Results:**

In the full analysis set (FAS), EP‐088_T502 significantly reduced the CSMS during the peak birch pollen season compared with placebo (mean absolute difference [MAD] −0.31; 95% confidence interval [CI], −0.56 to −0.05; *p* = 0.0169). In the per‐protocol (PP) and complete‐case sets, the corresponding MADs were −0.36 (*p* = 0.012) and −0.51 (*p* = 0.013), respectively. Health‐related QoL improved by 20% in the EP‐088_T502 group compared with placebo during the peak season (*p* < 0.005). EP‐088_T502 increased Bet v 1‐specific immunoglobulin G4 (IgG4) levels after treatment compared with placebo (6.4‐fold; *p* < 0.0001). The Bet v 1‐specific immunoglobulin E (IgE)/IgG4 ratio was reduced by 73% from baseline and by 69% compared with placebo (both *p* < 0.0001). Thirteen systemic allergic reactions occurred, including one grade III systemic reaction considered related to EP‐088_T502.

**Conclusions:**

EP‐088_T502 significantly improved symptom and medication scores in participants with birch pollen–induced ARC and induced a substantial Bet v 1‐specific IgG4 response. The 28,000 mTU regimen induced a substantial immunological response and showed an acceptable safety and tolerability profile.

**Abbreviations**
AE: adverse event
AIT: allergen immunotherapy
ANCOVA: analysis of covariance
ANOVA: analysis of variance
ARC: allergic rhinoconjunctivitis
CC: complete cases
CI: confidence interval
CSMS: Combined Symptom and Medication Score
DBPC: double‐blind placebo‐controlled
DC: dendritic cell
dMS: daily Medication Score
dSS: daily Symptom Score
DWD: German Weather Service (Deutscher Wetterdienst)
EAACI: European Academy of Allergy and Clinical Immunology
EPS: entire pollen season
FAS: full analysis set
GCP: Good Clinical Practice
IM: immunogenicity
IMP: Investigational Medicinal Product
IQR: interquartile range
LR: local reaction
MedDRA: Medical Dictionary for Regulatory Activities
mITT: modified intention‐to‐treat
mTU: mannan therapeutic units
PID: German Pollen Information Service (Polleninformationsdienst)
PP: per‐protocol set
PPS: peak pollen season
PRO: patient‐reported outcome
QoL: quality of life
RQLQ: Rhinoconjunctivitis Quality of Life Questionnaire
SAE: serious adverse event
SAF: safety analysis set
SCIT: subcutaneous immunotherapy
SD: standard deviation
SLIT: sublingual immunotherapy
SR: systemic reaction
TEAE: treatment‐emergent adverse event
Treg: regulatory T cell
V: visit

## 1 Introduction

Mannan‐conjugated allergoids have demonstrated efficacy, safety, and good tolerability as short‐course pre‐seasonal treatment for birch pollen allergy, as well as for grass pollen and house dust mite allergy [1]. Modified allergoids coupled to mannan show enhanced uptake by antigen‐presenting cells, particularly dendritic cells (DCs), thereby promoting immunomodulatory immune responses [2]. Mannan targets C‐type lectin receptors expressed on DCs, facilitating allergen uptake and immune processing [3]. Structural modification of allergen proteins and enhanced bioavailability may allow administration of higher cumulative allergen doses without increasing allergenicity[4, 5].

These developments represent an important step towards improving the safety and tolerability profile of subcutaneous immunotherapy (SCIT), while maintaining clinical efficacy [6].

The maximal well‐tolerated concentration of EP‐088_T502 (10,000 mannan therapeutic units per millilitre (mTU/mL)) was established in a previous dose‐finding trial [7]. Subsequently, a pivotal double‐blind placebo‐controlled (DBPC) phase III trial demonstrated that short‐course pre‐seasonal SCIT with EP‐088_T502 at a cumulative dose of 23,000 mTU significantly improved CSMS, induced relevant immunologic responses, and showed a favourable safety profile [8].

The present confirmatory DBPC phase III trial aimed to further evaluate the efficacy, safety, tolerability, and immunologic effects of EP‐088_T502 administered over six pre‐seasonal treatment visits at a cumulative dose of 28,000 mTU. The results were interpreted in the context of the previous dose‐finding and phase III trials of EP‐088_T502. In addition, efficacy and safety/tolerability were explored in adolescents.

## 2 Methods

### 2.1 Trial Design

The study was designed as a prospective, multicentre, double‐blind placebo‐controlled (DBPC) trial conducted in Germany and included 10 visits: one Screening visit, six treatment visits before the 2025 birch pollen season, two observational visits before and during the birch pollen season and one end of the trial visit after the birch pollen season (Figure 1). It was planned to randomise at least 273 participants (with 27 being adolescents) in a ratio of 1:2 (Placebo:EP‐088_T502) to the treatment.

**FIGURE 1:**
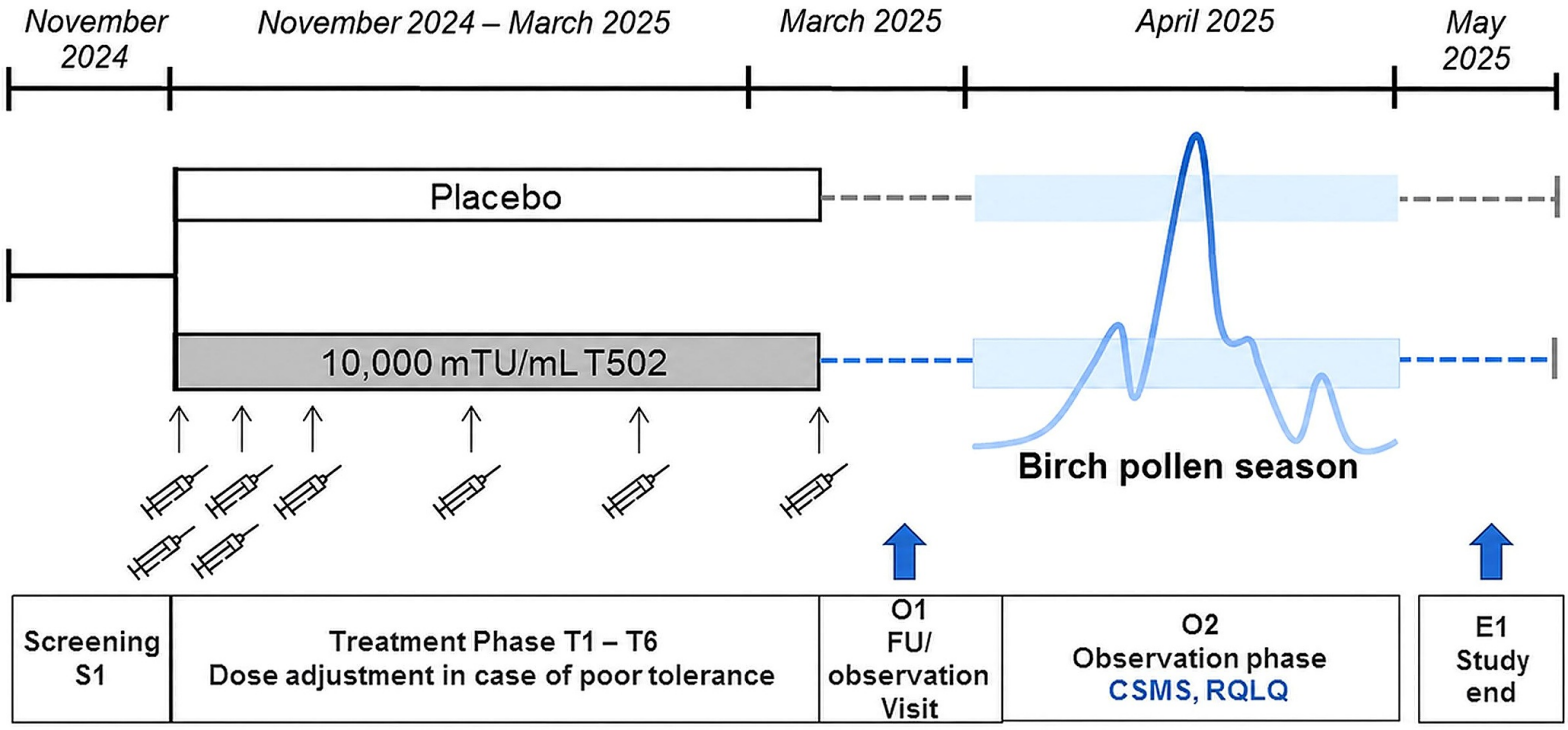
T502‐SIT‐073 trial design. Subcutaneous injections of EP‐088_T502 or placebo were administered before the start of the birch pollen season (treatment phase). The observation phase took place during the birch pollen season, during which CSMS and RQLQ data were collected to assess the clinical effect. CSMS, combined symptom and medication score; FU, follow‐up; mTU/mL, mannan therapeutic units per millilitre; RQLQ, Rhinoconjunctivitis Quality of Life Questionnaire.

For inclusion and non‐inclusion criteria, please refer to Supporting Information S1: Material S1.

### 2.2 Ethics Declaration

The trial was conducted in accordance with the Declaration of Helsinki (75th World Medical Association General Assembly, Helsinki, Finland, October 2024), Good Clinical Practice (GCP) guidelines (CPMP/ICH/135/95), and applicable national drug and data protection regulations [9].

Relevant trial documents, including participant information sheets, informed consent forms, and data protection declarations, were approved by the responsible ethics committees before trial initiation.

The Paul‐Ehrlich‐Institut, Germany, approved the trial (EU trial number: 2024‐515717‐17‐00) on 17 October 2024.

### 2.3 Allergen Immunotherapy (AIT)

Allergen immunotherapy (AIT) was performed using a mannan‐conjugated allergoid preparation derived from *Betula pendula* pollen allergens (EP‐088_T502, 10,000 mTU/mL) or placebo. Coupling of polymerised allergoids to mannan by glutaraldehyde treatment results in high‐molecular‐weight glycoconjugates with modified allergenic properties. Details regarding the manufacturing process have been described previously [3, 4, 5].

Participants received eight subcutaneous injections of EP‐088_T502 or placebo during six treatment visits before the start of the 2025 birch pollen season. The treatment schedule (Supporting Information S1: Material S2) included a short up‐dosing phase followed by a maintenance phase.

During the up‐dosing phase, treatment intervals between visits T1–T2 and T2–T3 ranged from 7 to 14 days. At T1, participants received injections of 0.1 mL followed by 0.2 mL if tolerated. At T2, injections of 0.2 and 0.3 mL were administered.

During the maintenance phase (T3–T6), 0.5 mL injections were administered at intervals of 21–35 days. Overall, participants received either placebo or a cumulative dose of 28,000 mTU EP‐088_T502 over a treatment period of 105 days.

### 2.4 Determination of the Pollen Season

The entire and peak birch pollen seasons were defined according to the EAACI recommendations for clinical trials in allergen immunotherapy published by Pfaar et al. [10].

Daily mean birch pollen concentrations (pollen/m^3^) used for the primary endpoint analysis were provided by the German Pollen Information Service (Polleninformationsdienst, PID), and each trial site was assigned to the nearest available monitoring station. If the peak birch pollen season identified for an individual trial site was shorter than 10 days, the continuous 14‐day period with the highest pollen load was used as the peak birch pollen season for that trial site (Supporting Information S1: Material S3). Because PID data were incomplete for several monitoring stations, a DWD‐based sensitivity analysis defined the peak birch pollen season as the 14 days in April with the highest forecast birch pollen levels in the respective geographical region.

### 2.5 Endpoints and Statistical Analysis

#### 2.5.1 Assessment of Efficacy—Combined Symptom and Medication Score

The CSMS was defined as the primary efficacy endpoint and was compared between the placebo and active treatment group during the peak birch pollen season 2025.

The CSMS combines daily symptom scores (dSS) and daily medication scores (dMS), each ranging from 0 to 3. Symptom severity was graded from no symptoms to severe symptoms, while medication use ranged from no medication to maximal permitted medication use [11]. An adapted version of the CSMS previously applied in earlier studies was used [8, 13].

The dSS included nasal symptoms (rhinorrhea, sneezing, nasal pruritus, nasal congestion) and ocular symptoms (ocular pruritus and watery eyes), each scored from 0 to 3 and averaged across six symptoms.

For the dMS, additional rescue medications beyond the classical EAACI 4‐step scoring system were permitted and scored additively as follows: antihistamine eye drops (0.5), oral antihistamines (1.0), and intranasal corticosteroids (1.5), resulting in a modified 7‐step scoring system ranging from 0 to 3.

Participants documented allergic symptoms and anti‐allergic medication use daily using the CCC STUDY Diary App. Both versions of the dMS have been shown to correlate well with a higher sensitivity to change for the additive score [12, 13].

#### 2.5.2 Assessment of Efficacy—Clinical Endpoints

Health‐related QoL was assessed using the Rhinitis Quality of Life Questionnaire (RQLQ) originally developed by Juniper and Guyatt [14], in its validated German version [15]. The RQLQ was designed for clinical trials to evaluate impairment associated with allergic rhinoconjunctivitis (ARC) symptoms. The questionnaire was completed before treatment initiation (T1), before the birch pollen season (O1), and during the birch pollen season (O2). Adolescent participants completed the AdolRQLQ.

To assess immunologic responses, serum levels of Bet v 1‐specific IgE, IgG, and IgG4 were measured before and after treatment (S1 and O1). Clinical laboratory analyses were performed at the central laboratory MLM Medical Labs (Mönchengladbach, Germany).

#### 2.5.3 Safety and Tolerability

Local reactions (LRs) (wheal and redness at the injection site) were assessed by investigators 30 minutes after each injection and documented as solicited local adverse events (AEs). Wheal diameters were categorised as mild, moderate, or severe according to immediate and delayed mean wheal diameters (< 5/< 10 cm, 5–10/10–20 cm, and > 10/> 20 cm, respectively).

In addition, other LRs and AEs, including SRs, were documented by participants on the evening of the injection day and during the following 2 days using the CCC STUDY Diary App. LRs other than wheals or redness, as well as all SRs, were recorded as unsolicited treatment‐emergent adverse events (TEAEs). All AEs were coded according to the Medical Dictionary for Regulatory Activities (MedDRA, version 25.0).

Vital signs and physical examinations were assessed at each visit.

Fexofenadine 180 mg was provided as rescue medication for on‐demand treatment of treatment‐related side effects during the treatment phase. Rescue medication use was documented in the treatment diary section of the CCC STUDY Diary App.

Blood samples for assessment of hematologic, renal, and liver function parameters were collected at S1, O1, and ET‐FU (if applicable).

In participants with asthma, pulmonary function testing by spirometry (FEV1) or peak expiratory flow (PEF) measurement was performed at each visit, particularly before and after treatment administration.

### 2.6 Statistical Analysis

Sample size calculations were performed using SAS for Windows version 9.4 based on results from the T502‐SIT‐045 trial. In the previous phase III trial (T502‐SIT‐045; EudraCT No. 2021‐002252‐36), the mean CSMS during the peak birch pollen season was 1.3 in the placebo group and 1.0 in the 10,000 mTU/mL treatment group within the modified intention‐to‐treat (mITT) population. The overall standard deviation (SD) was approximately 0.8.

Assuming a two‐sided alpha error of 5%, a statistical power of 80%, and a 1:2 randomization ratio (placebo:EP‐088_T502), the required sample size was calculated as 252 participants. After adjustment for an anticipated dropout rate of 7.5% after randomization, the final sample size was set at 273 participants (91 placebo and 182 active treatment participants). Approximately 400 participants were expected to be screened to compensate for screening failures.

The safety analysis set (SAF) and the full analysis set (FAS) included all randomised participants who received at least one dose of the investigational medicinal product (IMP) (*N* = 278; placebo, *n* = 92; EP‐088_T502, *n* = 186). The SAF was used for the safety analyses, whereas the FAS was used for the primary and secondary efficacy analyses. The modified full analysis set (mFAS) included treated participants with at least one post‐randomisation entry in the allergy diary (*N* = 262; placebo, *n* = 87; EP‐088_T502, *n* = 175). The per‐protocol set (PP) included evaluable participants who complied with the protocol according to criteria defined during the blinded data review and provided complete data for evaluation of the primary efficacy endpoint (*N* = 227; placebo, *n* = 76; EP‐088_T502, *n* = 151). The complete‐case set (CC) included participants in the FAS without missing CSMS values during the peak birch pollen season (*N* = 110; placebo, *n* = 42; EP‐088_T502, *n* = 68). The immunogenicity set included participants with at least two measurements of at least one immunological parameter (*N* = 267).

The primary efficacy analysis was conducted in the FAS within a prespecified estimand framework and estimated the between‐group difference in the participant‐level mean daily additive CSMS during the peak birch pollen season. Participants were analysed according to their assigned treatment group irrespective of treatment discontinuation. Use of prohibited rescue medication during the peak birch pollen season was handled using a prespecified composite strategy in the primary analysis.

For the primary analysis, missing daily CSMS values were assumed to be missing at random (MAR). The participant‐level mean daily CSMS was calculated using the available daily values. Where imputation was required, missing daily CSMS values were multiply imputed within the assigned treatment group using available CSMS data, including values recorded before an intercurrent event. The additive CSMS was imputed as a whole rather than through separate imputation of dMS and dSS. Estimates across the imputed datasets were combined using Rubin's rules.

The participant‐level mean daily CSMS during the peak birch pollen season was analysed using an analysis of variance (ANOVA) model with treatment group as a fixed effect and pooled trial site as a covariate. For all other CSMS, dMS, and dSS comparisons reported in Table 1, *p*‐values were obtained using the two‐sided Wilcoxon rank‐sum (Mann–Whitney U) test. Trial sites assigned to the same pollen‐monitoring station were pooled. The treatment effect was reported as the difference in mean daily CSMS between EP‐088_T502 and placebo, with a two‐sided 95% confidence interval and *p*‐value.

**TABLE 1:** (A) Combined symptom and medication score (CSMS), (B) daily medication score and (C) daily symptom score during the peak (PPS) and entire (EPS) birch pollen season for the full analysis set (FAS), PP‐set (PP) and the complete cases set (CC).

Pollen season	Analysis set	Descriptive statistics	Treatment group		*p*‐value
			Placebo	EP‐088_T502	
PPS	FAS	Mean	1.67	1.36	0.017
		SD	1.05	0.98	
		Median	1.49	1.23	
		Min/Max	0.00/4.63	0.00/5.19	
PPS	PP	Mean	1.67	1.31	0.012
		SD	1.07	1.01	
		Median	1.45	1.12	
		Min/Max	0.00/4.63	0.00/5.19	
PPS	CC	Mean	1.63	1.12	0.013
		SD	1.00	0.79	
		Median	1.40	1.05	
		Min/Max	0.25/4.31	0.00/2.74	
EPS	FAS	Mean	1.59	1.29	0.016
		SD	1.00	0.89	
		Median	1.51	1.18	
		Min/Max	0.00/4.63	0.00/4.52	
EPS	PP	Mean	1.59	1.23	0.007
		SD	1.02	0.91	
		Median	1.46	1.05	
		Min/Max	0.00/4.63	0.00/4.52	
EPS	CC	Mean	1.56	1.02	0.003
		SD	0.95	0.71	
		Median	1.37	0.93	
		Min/Max	0.18/3.75	0.00/2.42	
PPS	FAS	Mean	0.81	0.62	0.062
		SD	0.77	0.65	
		Median	0.62	0.48	
		Min/Max	0.00/3.00	0.00/3.00	
PPS	PP	Mean	0.80	0.61	0.058
		SD	0.77	0.68	
		Median	0.61	0.40	
		Min/Max	0.00/3.00	0.00/3.00	
PPS	CC	Mean	0.80	0.50	0.062
		SD	0.80	0.53	
		Median	0.50	0.36	
		Min/Max	0.00/2.82	0.00/1.96	
EPS	FAS	Mean	0.77	0.57	0.026
		SD	0.72	0.58	
		Median	0.61	0.47	
		Min/Max	0.00/2.75	0.00/3.00	
EPS	PP	Mean	0.76	0.56	0.036
		SD	0.73	0.60	
		Median	0.59	0.43	
		Min/Max	0.00/2.75	0.00/3.00	
EPS	CC	Mean	0.77	0.45	0.029
		SD	0.75	0.46	
		Median	0.51	0.28	
		Min/Max	0.00/2.61	0.00/1.65	
PPS	FAS	Mean	0.86	0.75	0.021
		SD	0.45	0.55	
		Median	0.83	0.66	
		Min/Max	0.00/2.57	0.00/2.94	
PPS	PP	Mean	0.87	0.70	0.007
		SD	0.47	0.53	
		Median	0.80	0.58	
		Min/Max	0.00/2.57	0.00/2.94	
PPS	CC	Mean	0.83	0.62	0.013
		SD	0.38	0.46	
		Median	0.79	0.51	
		Min/Max	0.25/1.65	0.00/2.02	
EPS	FAS	Mean	0.82	0.72	0.038
		SD	0.43	0.51	
		Median	0.78	0.64	
		Min/Max	0.00/1.95	0.00/2.92	
EPS	PP	Mean	0.83	0.67	0.008
		SD	0.45	0.49	
		Median	0.79	0.58	
		Min/Max	0.00/1.95	0.00/2.92	
EPS	CC	Mean	0.80	0.57	0.004
		SD	0.38	0.42	
		Median	0.82	0.54	
		Min/Max	0.15/1.80	0.00/1.82	

*Note:* Except for the primary FAS analysis, *p*‐values were obtained using the Wilcoxon rank‐sum (Mann–Whitney U) test. The primary FAS *p*‐value was obtained from the prespecified ANOVA model with treatment group as a fixed effect and pooled trial site as a covariate.

Mean daily CSMS, dMS, and dSS values were calculated for each participant during the peak and entire birch pollen seasons. Supportive analyses of the primary endpoint were conducted in the mFAS, PP, and CC populations and for the entire birch pollen season. Sensitivity analyses assessed alternative approaches to missing‐data handling, including group‐mean imputation, worst‐case imputation, and tipping‐point analyses. Additional sensitivity analyses included the weighted EAACI CSMS defined by Pfaar et al. [11] and the DWD‐based peak‐season analysis described above.

Normality of continuous variables was assessed using the Shapiro–Wilk test and graphical methods. Categorical variables were compared using the chi‐square test or Fisher's exact test, as appropriate. Comparisons between time points were performed using paired t tests or Wilcoxon signed‐rank tests. All statistical tests were two‐sided, and *p* < 0.05 was considered statistically significant. No adjustment for multiple comparisons was applied; *p*‐values for secondary and exploratory analyses were considered nominal.

Safety outcomes were summarised descriptively in the SAF. Immunological parameters were summarised descriptively for each assessment time point. Serum levels, IgE/IgG4 ratios, and absolute and relative changes from baseline were calculated. Changes in health‐related quality of life assessed using the RQLQ and AdolRQLQ were analysed using analysis of covariance (ANCOVA).

Data are presented as median (interquartile range [IQR]) and/or mean ± SD. Statistical analyses were performed using SAS for Windows version 9.4 and SPSS Statistics for Windows version 29.0.1 (IBM Corp., Armonk, NY, USA). Graphs were generated using GraphPad Prism version 10 for Windows (GraphPad Software, San Diego, CA, USA).

## 3 Results

### 3.1 General Trial Data and Baseline Characteristics

The trial was conducted between November 2024 (first screening visit) and June 2025 (last end‐of‐trial visit). The mean/median overall trial duration was 171.9/179.5 days in the placebo group and 169.8/179.0 days in the EP‐088_T502 group. Mean/median treatment duration was 101.3/104 days in the placebo group and 97.1/103 days in the EP‐088_T502 group.

Details regarding the duration of the entire and peak birch pollen seasons are provided in Supporting Information S1: Material S3.

Of the 376 screened participants (361 adults and 15 adolescents), 278 participants were randomised, including 13 adolescents (4.7%). Participants received either placebo (*n* = 92) or 10,000 mTU/mL EP‐088_T502 (*n* = 186) (Figure 2).

**FIGURE 2:**
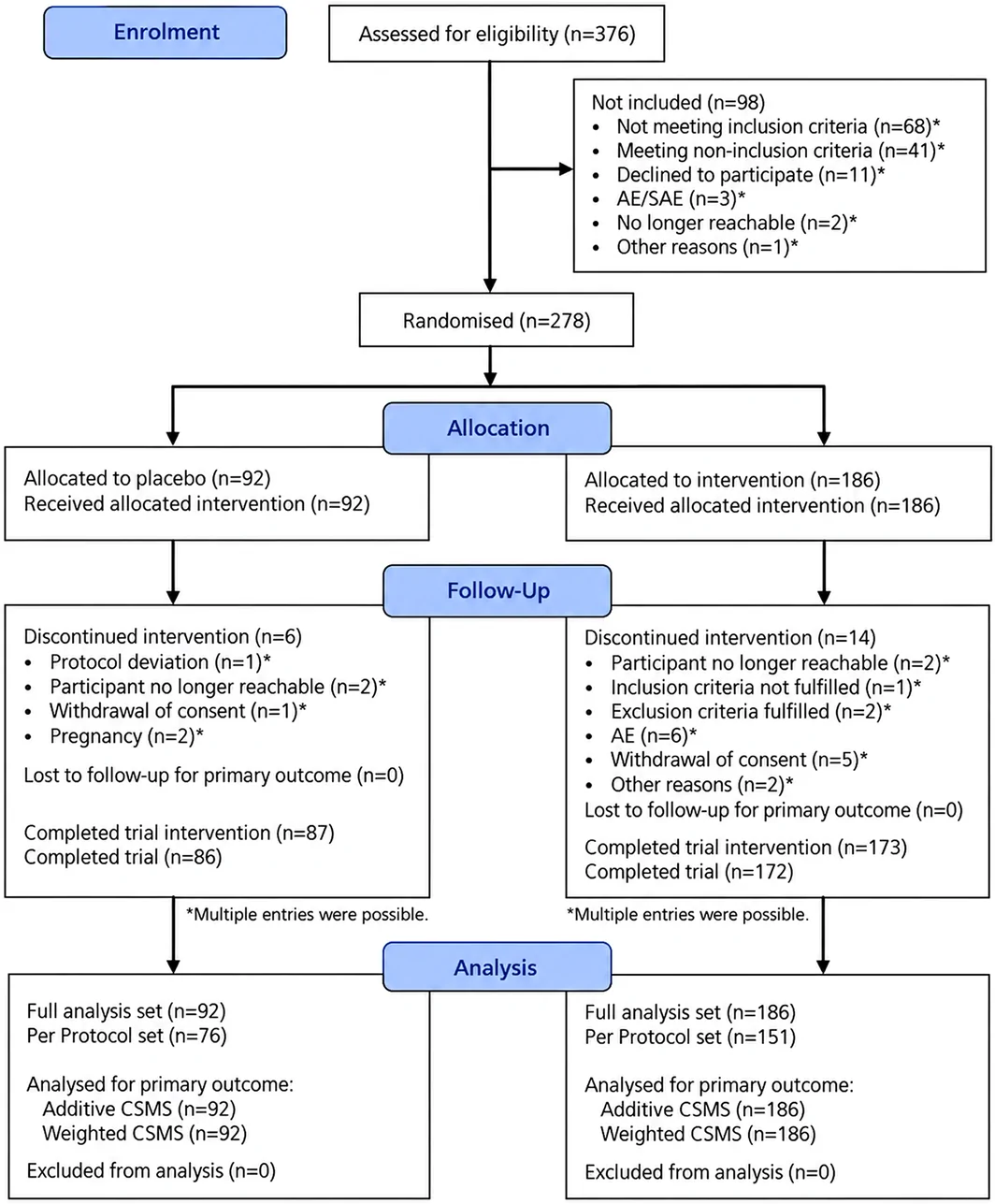
Flow chart of the trial.

Demographic characteristics, baseline clinical characteristics, and asthma status were comparable between treatment groups (Supporting Information S1: Material S4).

### 3.2 Allergy‐Related Baseline Values (SAF)

All randomised participants had a documented history of birch pollen‐induced ARC or birch pollen allergy for at least two birch pollen seasons and a positive skin prick test to birch pollen.

In the ARIA questionnaire, all participants (*n* = 278) reported troublesome rhinitis symptoms, and 242 participants (87.1%) reported persistent rhinitis symptoms (Supporting Information S1: Material S5). Baseline immunologic parameters were comparable between treatment groups. Mean birch pollen‐specific IgE levels (common silver birch, t3 serum level) measured at S1 were 25.00 kU/L in the placebo group and 29.92 kU/L in the EP‐088_T502 group. The distribution of CAP classes is shown in Supporting Information S1: Material Table S6.

### 3.3 CSMS During the Peak Birch Pollen Season

#### 3.3.1 Additive dMS, CSMS and dSS During the Peak Birch Pollen Season

In the FAS, the mean daily additive CSMS during the peak birch pollen season was lower in the EP‐088_T502 group than in the placebo group. The mean and median CSMS according to the adapted 7‐step score were reduced by 18.4% and 17.4%, respectively, compared with placebo. The absolute mean difference between treatment groups was −0.31 score points (95% confidence interval, CI, −0.56 to −0.05; *p* = 0.0169; Table 1A), corresponding to a standardised effect size of 0.31, which was below the proposed threshold of 0.5 [16, 17].

In the PP set, the absolute mean difference between treatment groups was −0.36 score points (*p* = 0.012). In the CC set, which included participants without missing CSMS values during the peak birch pollen season, the absolute mean difference was −0.51 score points (*p* = 0.013) (Table 1A).

Regarding medication use, the mean dMS during the peak birch pollen season was reduced from 0.81 in the placebo group to 0.62 in the EP‐088_T502 group, corresponding to an absolute difference of −0.19 score points and a median reduction of 22.4%. The between‐group difference did not reach statistical significance in the FAS (*p* = 0.062; Table 1B).

For symptom severity, the mean dSS during the peak birch pollen season was reduced from 0.86 in the placebo group to 0.75 in the EP‐088_T502 group. This corresponded to an absolute difference of −0.11 score points and a median reduction of 20.1% (*p* = 0.021; Table 1C).

Data regarding relative and absolute differences as well as standardised effect sizes for the CSMS, dSS and dMS are presented in Supporting Information S1: Material S7A.

### 3.4 dMS and CSMS According to EAACI During the Peak Birch Pollen Season

In addition to the adapted 7‐step CSMS used as the primary endpoint, the CSMS was also calculated according to the original EAACI/Pfaar definition as a pre‐specified sensitivity analysis.

Using the EAACI/Pfaar CSMS definition (Supporting Information S1: Material S7B), EP‐088_T502 was associated with lower symptom and medication scores compared with placebo during the peak birch pollen season. In the FAS, the mean and median absolute treatment effects were −0.27 and −0.32 score points, respectively, corresponding to relative reductions of 21.2% and 26.3%. The corresponding standardised effect sizes were 0.31 and 0.45, respectively.

In the PP set, the mean and median absolute differences were −0.32 and −0.44 score points, respectively. The corresponding relative reductions were 25.8% and 41.3%, with standardised effect sizes of 0.36 and 0.67, respectively.

For the EAACI‐defined dMS, the median reduction in the PP set during the peak birch pollen season was 68.2% compared with placebo, corresponding to a median‐based standardised effect size of 1.85. No use of oral glucocorticosteroids was documented during the trial, resulting in a maximum EAACI dMS of 2.

A treatment effect in the same direction was also observed across the entire birch pollen season. In the FAS, the mean and median absolute treatment effects for the EAACI‐defined CSMS were −0.26 and −0.35 score points, respectively, corresponding to relative reductions of 21.6% and 31.0%. The corresponding standardised effect sizes were 0.33 and 0.49, respectively.

Data regarding relative and absolute differences as well as effect sizes for the CSMS and dMS are displayed in Supporting Information S1: Material S7B.

#### 3.4.1 Sensitivity Analysis of Additive CSMS Using DWD Pollen Forecast Data

Because complete pollen‐count data were not available from several PID monitoring stations at the time of analysis, an additional sensitivity analysis was conducted in the FAS using daily regional birch pollen forecasts provided by the German Weather Service (Deutscher Wetterdienst, DWD).

Using the DWD‐based peak‐season definition, the mean CSMS was 1.701 in the placebo group and 1.357 in the EP‐088_T502 group. The estimated treatment difference was −0.34 score points (95% CI, −0.60 to −0.09; *p* = 0.0078), corresponding to an approximately 20% reduction compared with placebo (Supporting Information S1: Material S8).

### 3.5 Rhinoconjunctivitis Quality of Life Questionnaire (RQLQ)

Health‐related QoL was analysed in adults using the RQLQ and in adolescents using the AdolRQLQ at T1, O1, and O2.

At T1 (performed in winter), the mean RQLQ score in adults was 0.59 (SD: 0.88) in the placebo group and 0.59 (SD: 0.85) in the EP‐088_T502 group (*p* = 0.758).

At O1, the mean RQLQ score was 1.80 (SD: 1.06) in the placebo group and 1.48 (SD: 1.10) in the EP‐088_T502 group, corresponding to an 18% lower score in the EP‐088_T502 group compared with placebo (*p* < 0.05).

At O2, the mean RQLQ score was lower in the EP‐088_T502 group compared with placebo, with a reduction of 20% (*p* < 0.005) (Figure 3).

**FIGURE 3:**
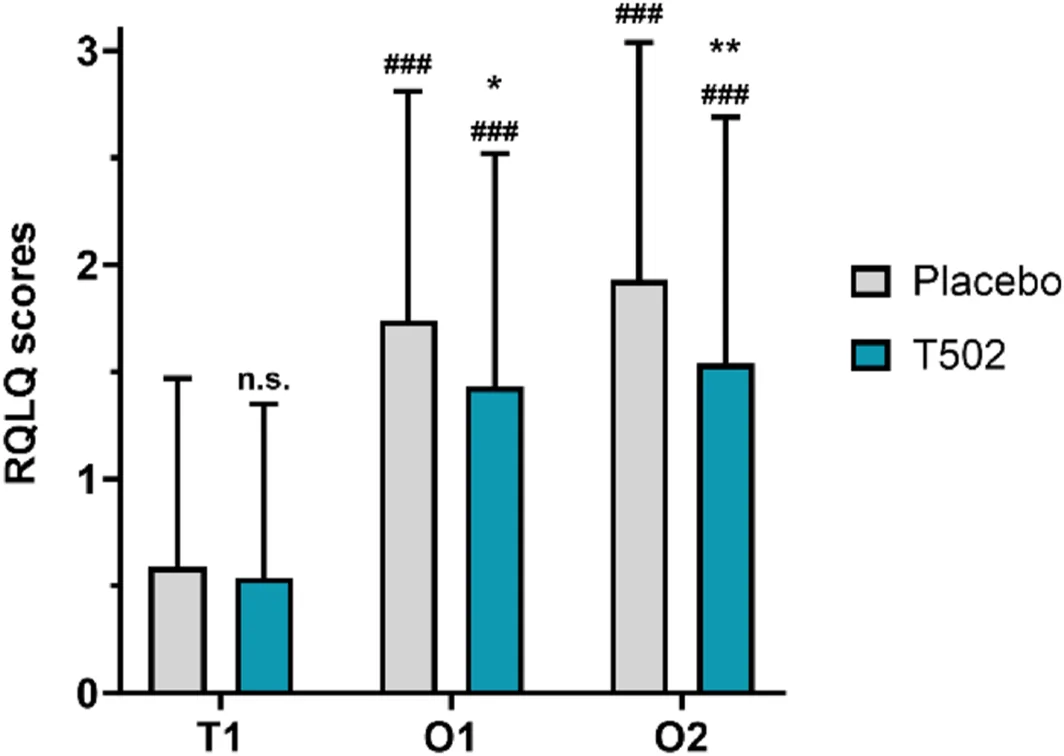
RQLQ scores before treatment (T1), before the birch pollen season (O1), and during the birch pollen season (O2). Data are presented as mean ± SD. *p < 0.05 compared with placebo; **p < 0.003; ###*p* < 0.0001 compared with T1; n.s., not statistically significant.

Regarding the adolescent subgroup, no statistically significant differences between treatment groups were observed at T1. In contrast to the findings in the overall and adult populations, changes from T1 to O2 in adolescents were also not statistically significant in the EP‐088_T502 group compared with placebo (*p* = 0.739; Supporting Information S1: Material S13).

### 3.6 Development of Immunological Parameters (Immunogenicity Set)

After treatment (O1), the mean Bet v 1‐specific sIgE level was 19.75 kU/L in the placebo group and 39.50 kU/L in the EP‐088_T502 group, corresponding to a 2.0‐fold higher level in the EP‐088_T502 group compared with placebo (*p* < 0.0001; Supporting Information S1: Material S9).

Subgroup analysis of adolescents showed slightly increased Bet v 1‐specific sIgE levels in the EP‐088_T502 group (1.2‐fold). Owing to the small sample size, these differences were not statistically significant (Supporting Information S1: Material S9).

Regarding Bet v 1‐specific sIgG4, values in the EP‐088_T502 group increased 6.0‐fold from S1 (0.50 mg/L) to O1 (3.01 mg/L), whereas sIgG4 levels in the placebo group remained largely unchanged. The between‐group difference in change from baseline was statistically significant (*p* < 0.0001).

In adolescents, treatment with EP‐088_T502 also increased the production of Bet v 1‐specific sIgG4 (5.4‐fold) compared with baseline. A 6.1‐fold increase was observed in adolescents compared with placebo after treatment. Owing to the small sample size in this subgroup, the differences were not statistically significant (Figure 4A).

**FIGURE 4:**
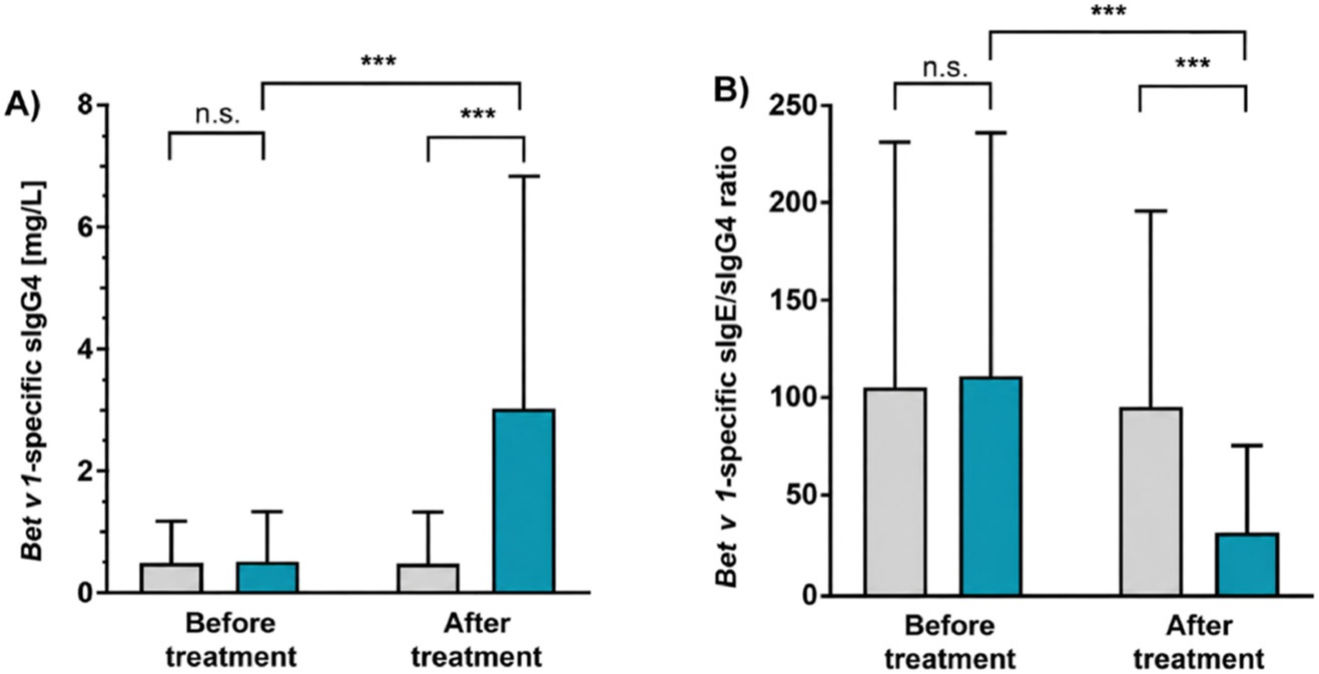
(A) Bet v 1‐specific sIgG4 levels before and after treatment with EP‐088_T502 or placebo. (B) Bet v 1‐specific sIgE/sIgG4 ratio before and after treatment with EP‐088_T502 or placebo. Data are presented as mean ± SD. *p < 0.05; ***p < 0.0001; n.s., not statistically significant. Grey bars indicate placebo, and blue bars indicate EP‐088_T502.

When comparing the sIgE/sIgG4 ratio at S1, mean values were similar between the placebo and EP‐088_T502 groups (105.31 and 110.59, respectively; n.s.). After treatment, the ratio decreased slightly in the placebo group (95.35). In the EP‐088_T502 group, the ratio was reduced by 73% (29.82). The between‐group difference in change from baseline was statistically significant (*p* < 0.0001).

In adolescents, the active treatment group also showed a reduction in the sIgE/sIgG4 ratio compared with baseline (64.7%, n.s.) (Figure 4B).

### 3.7 Safety Results

The analysis focussed on TEAEs. Overall, 537 TEAEs occurred in 138 participants (placebo: *n* = 27; EP‐088_T502: *n* = 111). For MedDRA‐based analyses, these TEAEs corresponded to 579 coded events, as individual TEAEs could be assigned to more than one MedDRA term.

The most common unsolicited TEAE in the active treatment group was injection‐site pruritus (*n* = 162, 32.73% of all TEAEs in this group [*n* = 495]), followed by injection‐site swelling (*n* = 73, 14.75%). The frequency distribution is shown in Supporting Information S1: Material S10.

In the EP‐088_T502 group, adolescent participants experienced a similar proportion of unsolicited LRs compared with adults (64.71% vs. 61.92%). In the placebo group, 7.14% of all TEAEs (*n* = 84) were unsolicited TEAEs and occurred exclusively in adults, with injection‐site pruritus (2.38%) being the most common event (Supporting Information S1: Material S10).

Three SAEs occurred during the trial. All SAEs occurred in adults, and all affected participants recovered without complications. One SAE was considered related to the IMP and was classified as a severe Grade III SR according to AWMF criteria. Narratives of this SR are provided in Supporting Information S1: Material S11.

Overall, 13 SRs occurred during the trial. Twelve SRs occurred in 11 participants treated with EP‐088_T502 (5.9%), whereas one Grade II SR occurred in one placebo‐treated participant. One Grade III systemic reaction occurred in a participant treated with EP‐088_T502. No Grade IV systemic allergic reactions according to the AWMF classification were reported. None of the participants experiencing SRs were adolescents or had asthma.

In relation to the number of EP‐088_T502 injections administered (*n* = 1608), SRs occurred in 0.75% of all injections.

In addition to TEAEs, local wheal reactions at the injection site were assessed. Nearly all wheal diameters measured 30 minutes after EP‐088_T502 injection were either 0 cm (35.3%) or < 5 cm (64.4%). Moderate wheal diameters accounted for 0.3% of measurements in the EP‐088_T502 group.

Among adolescent participants treated with EP‐088_T502 (*n* = 9), wheals were also either absent (0 cm, 48.6%) or mild in severity (< 5 cm, 51.4%) (Supporting Information S1: Material S12).

Regarding late‐phase local wheal reactions, wheals were absent (0 cm) in 95.7% of all measurements in the placebo group and in 69.5% of measurements in the EP‐088_T502 group. Of all documented wheals in the active treatment group (*n* = 3759), two (0.1%) were categorised as severe (wheal diameter > 20 cm).

## 4 Discussion

In adolescents, nearly all wheals in the placebo group (98.7%) measured 0 cm. In the active treatment group, most wheal diameters (65.2%) were 0 cm. One wheal in an adolescent participant treated with EP‐088_T502 was categorised as severe (> 20 cm) (Supporting Information S1: Material S12).

The aim of this confirmatory phase III trial was to further evaluate the efficacy, safety, tolerability, and immunological effects of short‐course SCIT with EP‐088_T502 administered over six pre‐seasonal treatment visits at a cumulative dose of 28,000 mTU. The findings were considered in the context of the two previous EP‐088_T502 trials, including the dose‐finding trial and the T502‐SIT‐045 phase III trial. In addition, efficacy and safety/tolerability were explored in adolescents.

In the current trial, the median CSMS in the active treatment group was reduced by 17.4% compared with placebo using the pre‐specified adapted 7‐step score in the FAS during the peak birch pollen season. When calculated according to the EAACI/Pfaar definition for the peak birch pollen season, the median relative reduction in CSMS was 26.3% in the FAS and 41.3% in the PP set. The median dMS was reduced by 22.4% using the adapted score in the FAS and by 68.2% using the EAACI definition in the PP set, whereas the median dSS was reduced by 20.1% in the FAS. These findings indicate reduced medication use and fewer symptoms during the birch pollen season and are consistent in direction with the results of the two previous EP‐088_T502 trials. In the T502‐SIT‐045 trial, the median CSMS in the active treatment group was reduced by 33%, the dSS by 30.4%, and the dMS by 56.3% compared with placebo [8]. In the earlier dose‐finding trial, participants treated with 10,000 mTU/mL EP‐088_T502 showed a median CSMS reduction of 24.7% compared with placebo [7]. However, direct numerical comparison of the treatment‐effect estimates across the three trials is limited by differences in study design, analysis populations, endpoint definitions, pollen seasons, and approaches to missing outcome data.

In the present trial, the primary treatment effect was estimated in the FAS within a pre‐specified estimand framework, with missing daily CSMS values handled using multiple imputation under the missing‐at‐random assumption. In contrast, the previous phase III trial used an intention‐to‐treat/evaluable‐data approach based on the availability of CSMS diary data during the peak birch pollen season. These approaches address related but not identical clinical questions. More generally, numerical comparisons of treatment effects across allergen immunotherapy trials should be interpreted in light of the estimand, analysis population, handling of intercurrent events, and missing‐data strategy used in each study. Consequently, differences in the numerical magnitude of the reported treatment effects should not be interpreted as evidence that the FAS analysis underestimated the treatment effect or as evidence of a dose‐dependent difference in efficacy.

The present trial adds evidence for the clinical efficacy of EP‐088_T502 administered over six pre‐seasonal treatment visits at a cumulative dose of 28,000 mTU and additionally provides an off‐season QoL baseline and exploratory data in adolescents.

Beyond the adapted 7‐step CSMS used as the primary endpoint, a treatment effect in the same direction was also observed when the original EAACI/Pfaar CSMS was applied as a pre‐specified sensitivity analysis. Under this scoring system, the standardised effect size of EP‐088_T502 on CSMS during the peak pollen season was 0.31 (mean‐based) and 0.45 (median‐based) in the FAS, with corresponding values of 0.36 and 0.67 in the PP set. The standardised effect sizes therefore ranged from 0.31 to 0.67, with only the median‐based PP estimate exceeding the proposed threshold of 0.5; overall, the estimates were consistent with effect sizes reported for other licensed birch pollen products. In the PP set during the peak pollen season, the median dMS was reduced by 68.2% in the EP‐088_T502 group compared with placebo, with a standardised effect size of 1.85. No participant required oral glucocorticosteroids during the trial; consequently, the maximum observed EAACI dMS was 2.

The treatment effect was assessed across several complementary analyses of the primary efficacy endpoint. In addition to the primary analysis in the full analysis set (mean treatment effect −0.31, *p* = 0.0169), treatment‐effect estimates were obtained in the per‐protocol set (−0.36, *p* = 0.0117) and in participants with complete diary records during the peak pollen season (−0.51, *p* = 0.0129) (Figure 5A). Official PID pollen‐count data were incomplete for several monitoring stations at the time of analysis. The DWD‐based analysis was therefore conducted as an additional sensitivity analysis to assess the treatment effect when the peak‐season window was defined using an alternative data source. The resulting treatment difference (−0.34; 95% CI, −0.60 to −0.09; *p* = 0.0078) was similar to that observed in the primary PID‐based analysis (−0.31; 95% CI, −0.56 to −0.05; *p* = 0.0169).

**FIGURE 5:**
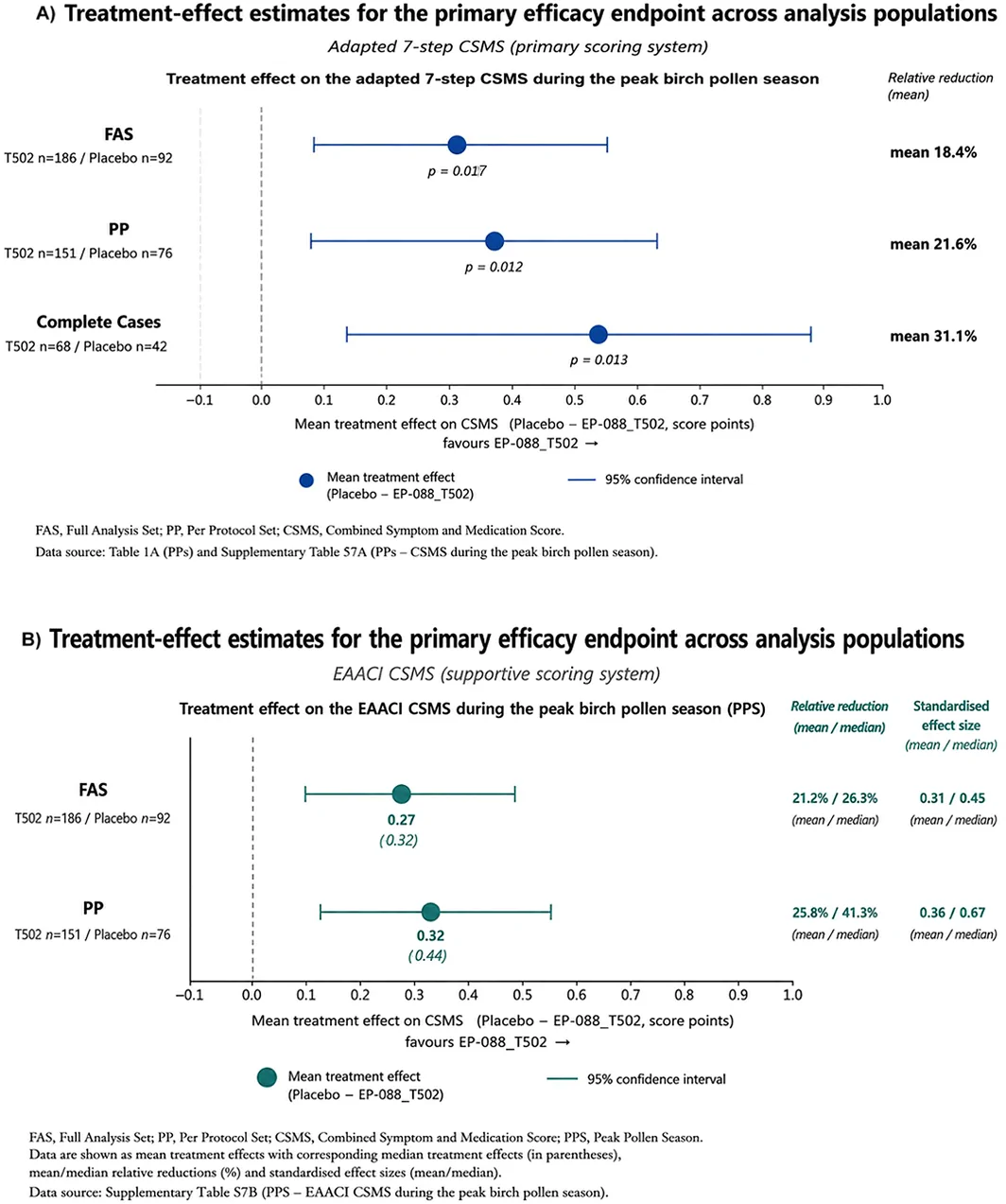
Forest plots of treatment‐effect estimates during the peak birch pollen season for the adapted 7‐step CSMS (A; FAS, PP, and complete‐case sets) and the EAACI/Pfaar CSMS (B; FAS and PP sets). CSMS, combined symptom and medication score; FAS, Full Analysis Set; PP, Per Protocol.

The analysis based on the original EAACI/Pfaar CSMS definition also showed a treatment effect in the same direction during the peak birch pollen season (mean absolute difference −0.27 in the FAS; standardised effect size 0.45 in the median‐based FAS analysis and 0.67 in the median‐based PP analysis; Figure 5B). These complementary analyses differed with respect to the analysis population, completeness of outcome data, score definition, or definition of the peak pollen season. Their estimates should therefore be interpreted as supportive findings rather than as estimates of an identical treatment effect or as evidence that the primary FAS estimate was attenuated. Unlike the CSMS, Bet v 1‐specific sIgG4 was measured at fixed visits and was therefore independent of the definition of the peak pollen window. Thus, the numerically greater immunological response but smaller clinical effect compared with T502‐SIT‐045 should be interpreted cautiously and does not by itself establish either reduced biological activity or a dose–response relationship.

Taken together, the statistically significant result of the primary FAS analysis, the treatment effects observed using the EAACI/Pfaar CSMS, and the consistent direction of the dSS and dMS components support the conclusion that the efficacy criteria for allergen immunotherapy products formulated by the Paul‐Ehrlich‐Institut were met.

Clinical efficacy of allergen immunotherapy assessed by CSMS reduction has also been demonstrated in other studies. Pfaar et al. [18] reported a 32% reduction in CSMS during the birch pollen season following treatment with a sublingual birch pollen preparation. In another study by Pfaar et al., [19] depigmented‐polymerised mixed grass/birch pollen SCIT reduced CSMS values by 33.7% in the active treatment group during the second treatment year compared with placebo.

The beneficial effects of EP‐088_T502 treatment were not limited to CSMS improvement but were also reflected in health‐related QoL outcomes. In contrast to the previous T502‐SIT‐045 trial, in which the RQLQ was assessed only immediately before and during the birch pollen season [8], the present trial additionally included an assessment at treatment initiation during autumn/winter, providing an off‐season baseline obtained before the main tree pollen season.

Assessment of the RQLQ outside the birch pollen season appears particularly relevant, since scores measured immediately before the birch pollen season were already almost twice as high as the autumn/winter baseline values, possibly reflecting exposure to alder and hazel pollen. Consequently, differences between pre‐seasonal and peak‐season measurements become less pronounced. These findings also support observations from the previous T502‐SIT‐045 study, in which RQLQ assessment immediately before the birch pollen season was likely influenced by early tree pollen exposure, limiting comparison with an off‐season baseline measurement. In adolescents, only marginal improvements in RQLQ scores were observed; however, interpretation is limited by the small sample size (*n* = 4 placebo; *n* = 9 EP‐088_T502).

As patient‐reported outcomes such as symptom diaries and questionnaires are inherently influenced by subjective symptom perception, objective immunological parameters were additionally assessed. Bet v 1‐specific sIgE increased 1.4‐fold from baseline in the EP‐088_T502 group and was 2.0‐fold higher than in the placebo group after treatment. A comparable increase was observed in the previous T502‐SIT‐045 trial, in which mean Bet v 1‐specific sIgE increased from 22.15 kU/L at baseline to 37.42 kU/L after treatment and subsequently decreased slightly to 35.11 kU/L at the end of the trial [8]. Thus, a similar sIgE increase was observed in both trials; however, this cross‐trial comparison does not permit conclusions about the effect of cumulative dose. The mechanism and clinical relevance of this increase cannot be determined from the present study.

At the same time, Bet v 1‐specific sIgG4 levels were 6.4‐fold higher after treatment with EP‐088_T502 compared with placebo (*p* < 0.0001). In the previous T502‐SIT‐045 trial, Bet v 1‐specific sIgG4 levels were 5.2‐fold higher after treatment with EP‐088_T502 compared with placebo. Although the sIgG4 response was numerically greater in the present trial, this comparison was made between separate trials rather than between randomised dose groups and therefore does not establish a dose–response relationship. Similarly, the numerically greater immunological response accompanied by a smaller clinical treatment‐effect estimate should not be interpreted as evidence of a direct relationship between the magnitude of the immunological and clinical effects. Accordingly, the clinical benefit of further dose escalation cannot be inferred from the present study and would require evaluation in a dedicated dose‐comparison trial.

In both studies, the development of immunological parameters supports the concept that short‐course treatment can induce relevant immunological effects. Consequently, the sIgE/sIgG4 ratio was also markedly reduced after treatment compared with placebo.

Treatment adherence remains a challenge in allergen immunotherapy [21, 22]. Completion rates were high in both the previous trial (91%) and the present trial (93%; *n* = 258), supporting the feasibility of the short‐course schedule. Regarding safety, SCIT is generally considered to be associated with a higher frequency of SRs than SLIT. However, besides the up‐dosing schedule, the use of native allergen extracts has also been associated with an increased risk of SRs during AIT. The present trial provides further safety and tolerability data for short‐course mannan‐conjugated SCIT.

In the present trial, 13 systemic allergic reactions (Grades I–III) occurred. Twelve SRs occurred in 11 of 186 participants treated with EP‐088_T502 (5.9%), and one Grade II SR occurred in a placebo‐treated participant. One treatment‐related Grade III SR occurred in an adult, non‐asthmatic participant and resolved within 6 hours. No Grade IV SRs were reported, and none of the participants experiencing an SR were adolescents or had asthma. Although the Grade III event is clinically relevant and underscores the need for appropriate precautions during SCIT administration, the overall safety and tolerability profile remained acceptable. These findings are consistent with the safety results of the previous trial, in which 16 SRs (Grade I/II) were reported. Wheal diameters measured 30 minutes after injection were absent or mild in most participants in both studies, which is in line with the tolerability profile described for other SCIT preparations. According to previous studies, the incidence of SRs per injection visit generally remains below 1% [23, 24, 25].

In relation to the number of participants treated with EP‐088_T502, SRs occurred in 5.9% of participants in the present trial. In the previous T502‐SIT‐045 trial, the proportion was slightly higher, with 8.1% of participants experiencing at least one SR. Calderon et al. [26] reported frequencies ranging from 22% for immediate systemic reactions to 89% for late‐phase systemic reactions in a Cochrane systematic review of SCIT [26]. The low number of treatment discontinuations in the present trial provides additional information on the overall tolerability of the short‐course regimen.

To minimise the risk of AEs and SRs, EAACI recommendations for safe AIT administration were followed [27]. Emergency equipment and trained physicians for anaphylaxis management were available at all trial sites. In addition, participants remained under observation for 30 minutes after each injection, since SRs frequently occur during this period [28]. Nevertheless, delayed SRs occurring after 30 minutes have also been reported, with some studies describing delayed reactions in 50% or more of cases [25, 28]. In the present trial, delayed SRs also accounted for a relevant proportion of cases.

Preventive measures are particularly important in asthmatic participants, as asthma may represent one of the main risk factors for SRs during AIT. To ensure participant safety, asthma control and pulmonary function were closely monitored throughout the trial. These findings underline the importance of assessing asthma control status before and during treatment, while also demonstrating that well‐controlled asthma does not necessarily represent a contraindication to AIT [29].

One limitation of the present trial is the relatively small adolescent subgroup. Although 10% adolescent recruitment had originally been planned, only 4% of participants were adolescents. Given the small subgroup size, no definitive conclusions can be drawn regarding treatment outcomes in adolescents. The findings in this subgroup should therefore be considered exploratory and require confirmation in larger paediatric studies. Large paediatric allergen immunotherapy trials have also been conducted in other allergen settings [20].

In conclusion, EP‐088_T502 demonstrated efficacy as a short‐course treatment and showed an acceptable safety and tolerability profile. The findings were consistent in direction with those of previous EP‐088_T502 studies. However, numerical comparisons of treatment effects across these trials should be interpreted in the context of differences in estimands, analysis populations, endpoint definitions, and approaches to missing outcome data. The present study does not establish a clinical benefit of increasing the cumulative dose, which would require evaluation in a dedicated randomised dose‐comparison trial.

## Author Contributions

**Esther Raskopf:** conceptualization, investigation, writing – original draft, writing – review and editing, supervision, project administration. **Sofia Passamera:** formal analysis, data curation, writing – review and editing, visualization, project administration. **Ludger Klimek:** methodology, validation, investigation, funding acquisition. **Oliver Pfaar:** conceptualization, methodology, validation, writing – review and editing. **Christian Neuhof:** validation, data curation, writing – original draft, project administration. **Anna Rybachuk:** validation, writing – original draft, writing – review and editing, visualization. **Nadine Katzke:** data curation, writing – original draft, writing – review and editing, visualization, project administration. **Hacer Sahin:** methodology, validation, formal analysis, data curation, writing – review and editing. **Silke Allekotte:** conceptualization, validation, resources, writing – review and editing, supervision, project administration, funding acquisition. **Ralph Mösges:** conceptualization, formal analysis, resources, supervision, writing – original draft, writing – review and editing. **Laura Day:** validation, writing – review and editing, project administration. **José Luis Subiza:** conceptualization, validation, resources, writing – review and editing, supervision, funding acquisition. **Miguel Casanovas:** conceptualization, validation, resources, writing – review and editing, funding acquisition. **Mandy Cuevas:** conceptualization, formal analysis, investigation, writing – review and editing, supervision. **Sandra del Pozo:** conceptualization, validation, writing – review and editing, supervision, project administration. All authors have read and approved the final version of the manuscript.

## Funding

Article processing charges were funded by Inmunotek S.L. All authors approved the final version of the manuscript before submission.

## Ethics Statement

The trial was conducted in accordance with the Declaration of Helsinki, Good Clinical Practice (GCP) guidelines, and applicable national regulations. The study was approved by the Paul‐Ehrlich‐Institut, Germany (EU trial number: 2024‐515717‐17‐00), and by the responsible ethics committees before trial initiation.

## Conflicts of Interest

E.R., C.N., A.R., N.K., H.S., S.A. and L.D. are employees of ClinCompetence Cologne GmbH and have no conflicts of interest to disclose. S.P. has nothing to disclose. L.K. reports grants and personal fees from Inmunotek during the conduct of the trial; grants and personal fees from Allergopharma, grants and personal fees from Viatris, personal fees from HAL Allergie, personal fees from ALK‐Abelló, grants and personal fees from LETI Pharma, grants and personal fees from Stallergenes, grants from Quintiles, grants and personal fees from Sanofi, grants from ASIT biotech, grants bromoform, personal fees from Allergy Therapeutics, grants from AstraZeneca, grants and personal fees from GSK, grants from Inmunotek, personal fees from Cassella med, personal fees from Novartis, personal fees from Regeneron Pharmaceuticals, personal fees from ROXALL Medizin GmbH, outside the submitted work; and Membership: AeDA, DGHNO, Deutsche Akademie für Allergologie und klinische Immunologie, HNO‐BV, GPA, EAACI. OP reports grants for his institution during the conduct of the trial from Inmunotek S.L., Spain; he also reports grants and/or personal fees and/or travel support from AEDA, Alfried Krupp Krankenhaus, ALK‐Abelló, Allergopharma, Almirall, Altamira Therapeutics, ASIT Biotech, AstraZeneca, Bencard Allergie GmbH/Allergy Therapeutics, Blueprint, Breazy Health, Cliantha, Deutsche AllergieLiga e.V., Deutsche Forschungsgemeinschaft, Dustri‐Verlag, ECM Ex‐pro&Conference Management GmbH, Forum für Medizinische Fortbildung, Georg‐Thieme‐Verlag, GSK, HAL Allergy Holding B.V./HAL Allergie GmbH, Inmunotek, Ingress Health, Institut für Disease Management, IQVIA Commercial, Japanese Society of Allergology, Königlich Dänisches Generalkonsulat, Laboratorios LETI/LETI Pharma, Lilly, Lofarma, Medizinische Hochschule Hannover, med update Europe GmbH, Meinhardt Congress GmbH, Novartis, Paul‐Ehrlich‐Institut, Paul‐Martini‐Stiftung, PneumoLive, Pohl‐Boskamp, Procter & Gamble, Red Maple Trials Inc., Regeneron, RG Aerztefortbildung, ROXALL Medizin, Sanofi Aventis, Sanofi Genzyme, Springer Publisher, Stallergenes Greer, streamedup! GmbH, Technical University Dresden, John Wiley & sons publishers, Wort & Bild Verlag, Verlag ME, all outside the submitted work. In addition, OP is Vice President of the European Academy of Allergy and Clinical Immunology (EAACI), a member of EAACI Excom and a member of the external board of directors of the German Society of Allergy and Clinical Immunology (DGAKI); the coordinator, main author, or co‐author of different position papers and guidelines in rhinology, allergology, and allergen immunotherapy; and the Editor‐in‐Chief of Clinical Translational Allergy and Associate Editor of Allergy. R.M. reports grants and personal fees from Inmunotek during the conduct of the trial; personal fees from ALK, grants from ASIT biotech, personal fees from Allergopharma, personal fees from Allergy Therapeutics, grants and personal fees from Bencard, grants from Leti, grants, personal fees and non‐financial support from Lofarma, non‐financial support from Roxall, grants and personal fees from Stallergenes, grants from Optima, personal fees from Friulchem, personal fees from Hexal, personal fees from Servier, personal fees from Klosterfrau, non‐financial support from Atmos, personal fees from Bayer, non‐financial support from Bionorica, personal fees from FAES, personal fees from GSK, personal fees from MSD, personal fees from Johnson & Johnson, personal fees from Meda, personal fees and non‐financial support from Novartis, non‐financial support from Otonomy, personal fees from Stada, personal fees from UCB, non‐financial support from Ferrero, grants from Hulka, personal fees from Nuvo, grants and personal fees from Ursapharm, personal fees from Menarini, personal fees from Mundipharma, personal fees from Pohl‐Boskamp, grants from Cassella‐med GmbH & Co. KG, personal fees from Laboratoire de la Mer, personal fees from Sidroga, grants and personal fees from HAL BV, personal fees from Lek, personal fees from PRO‐AdWise, personal fees from Angelini Pharma, grants and non‐financial support from JGL, grants and personal fees from bitop, grants from Sanofi, outside the submitted work; MC declares honoraria for presentations ALK‐Abelló, Allergopharma, AstraZeneca, Bencard Allergie/Allergy Therapeutics, Celltrion Healthcare Deutschland GmbH, GlaxoSmithKline, HAL Allergy, Leti Pharma, NeilMed, Novartis, Roxall, Sanofi‐Aventis, Stallergenes outside the submitted work. Other non‐financial interests: German Society of Allergy (AeDA), German Society of Oto‐Rhino‐Laryngology, Head and Neck Surgery DGHNO‐KHC. M.C. is the coordinating investigator of the presented clinical trials. JLS and MCV are shareholders of Inmunotek, SdP is an employee of Inmunotek. All authors had full access to all the data in this trial and take complete responsibility for the integrity of the data and accuracy of the data analysis.

## Supporting information


Supporting Information S1

## Data Availability

The data that support the findings of this study are available on request from the corresponding author. The data are not publicly available due to privacy or ethical restrictions.
