# Supplementary material for: Short‐Course Subcutaneous Immunotherapy With Mannan‐Conjugated Birch Pollen Allergoids: Consistent Clinical and Immunological Improvements
Source: Clin Transl Allergy. 2026 Oct 4;16(10):e70209. doi: 10.1002/clt2.70209 (PMC13635281; doi:10.1002/clt2.70209)
Supplement: Supplementary file 1 [file CLT2-16-e70209-s001.docx]

**Supplementary Material**

**Supplementary Material S1.** Inclusion and non-inclusion criteria of the T502-SIT-073 trial

# Inclusion criteria

## Inclusion Criteria

The inclusion criteria had to be checked at S1 and to be rechecked at T1 before starting the treatment to confirm eligibility.

Participants had to meet all of the following inclusion criteria in order to participate in this trial:

1. Participants aged from 12 to 64 years
2. Signed and dated Informed Consent Form
   a. by a legally competent participant,
   b. For adolescents: self-completed (signed and dated) informed consent to participate in the trial and signed and dated Informed Consent Form by both parents/legal guardian(s)
3. Being in good physical and mental health,
4. Having the diagnosis of birch pollen allergy based on all the following criteria:
   a. A medical history of moderate to severe allergic rhinitis or rhinoconjunctivitis due to birch pollen allergens for at least 2 previous seasons (definition of allergy severity according to ARIA),
   b. Being treated with anti-allergic medication for at least 2 birch pollen seasons prior to enrolment,
   c. A positive skin prick test (SPT - wheal diameter ≥3 mm) to birch pollen allergens, positive control (histamine) wheal ≥3 mm, negative control (NaCl) wheal <2 mm.
5. Females
   a. With childbearing potential (a woman is considered of childbearing potential [WOCBP] according to the CTFG, if she is i.e., fertile, following menarche and until becoming postmenopausal unless becoming permanently sterile. Permanent sterilisation methods include hysterectomy, bilateral salpingectomy and bilateral oophorectomy) had to be willing to use a highly effective method of contraception:
      - Oral, intravaginal or transdermal hormonal medical drugs or -devices containing oestrogen/progesterone combinations.
      - Oral, injectable or implantable hormonal medical drugs or -devices containing progesterone-only.
      - Intrauterine device (IUD).
      - Intrauterine hormone-releasing system (IUS).
      - Bilateral tubal occlusion.
      - Vasectomized partner (provided that partner was the sole sexual partner of the WOCBP trial participant and that the vasectomized partner had received medical assessment of the surgical success).
      - Sexual abstinence (Defined as refraining from heterosexual intercourse during the entire period of risk associated with the trial treatments. The reliability of sexual abstinence needed to be evaluated in relation to the duration of the clinical trial and the preferred and usual lifestyle of the participant).
   b. Females not of childbearing potential (i.e., pre-menarche, tubal ligation, hysterectomy, or post-menopausal (a postmenopausal state is defined as no menses for 12 months without an alternative medical cause.),
6. For asthmatic participants: confirmed diagnosis of controlled asthma during the treatment period according to Global Initiative for Asthma (GINA) guidelines (steps 1-3, GINA 2023),
7. FEV1≥80% of the participant’s reference value or Peak Expiratory Flow (PEF) ≥80% of the participant’s individual optimal value (for asthmatic participants only) measured at the screening visit.
8. Laboratory tests
   a. Specific IgE against birch pollen allergens (common silver birch t3, minimum(CAP) class 3 or higher, ≥3.5 kU/L); in case that the IgE CAP-class was = 2, the participant could have been included, when a previous lab report (not older than 2 years and measurement outside the birch pollen season) stated that the participant had CAP class 3 or higher)
   b. Confirmed normal renal and liver function, including non-clinically significant deviations outside the reference ranges (< grade 2 according to the Food and Drug Administration (FDA) Guidance for Industry for preventive Vaccine Trials [FDA 2007] at screening visit. Participants with laboratory values ≥ grade 2 required retesting before inclusion in the trial. Upon normalization of the out-of-range value(s), the participant could have been included in the trial),
   c. Female participants with childbearing potential had to have a negative pregnancy test in serum at screening.

## **Non-inclusion criteria**

The exclusion criteria had to be checked at S1 and to be rechecked at T1 before starting the treatment to confirm eligibility.

At screening, participants were not eligible if any of the following exclusion criteria were met in order to participate in this trial.

1. Simultaneous participation in other clinical trials or previous participation within 30 days before inclusion,
2. Previous immunotherapy with birch pollen allergens within the last 5 years,
3. Ongoing immunotherapy with birch pollen allergens or any other allergens,
4. Participants with acute allergic rhinitis or rhinoconjunctivitis due to other environmental allergens during the trial period,
5. Being in any relationship or dependence with the Sponsor, CRO and/or Investigator,
6. Inability to understand instructions/trial documents,
7. Participants for whom the Investigator believes that they would not comply with the protocol (participants with known alcohol or drug abuse or with a history of a serious psychiatric disorder as well as participants unwilling to give informed consent or to abide by the requirements of the protocol).
8. Participants who were committed to an institution by virtue of an order issued either by the judicial or the administrative authorities.
9. Participants who did not have access to a smartphone/tablet (iOS or Android, in exceptional cases, a paper diary could be issued if installation on the mobile device was not technically possible),
10. History of systemic reactions and/or anaphylaxis (Grade III or IV according to the AWMF guideline 2021), including to food (e.g., peanut, marine animals) or to Hymenoptera venom (e.g., bee, wasp stings) or to medication (e.g., penicillin), etc.,
11. History of hypersensitivity to the excipients of the investigational product or placebo,
12. Mild persistent to severe persistent asthma, partly controlled or uncontrolled asthma according to GINA guidelines (GINA 2023) during the treatment period,
13. Chronic asthma or emphysema, particularly with a Forced Expiratory Volume in 1 second (FEV1) <80% of the participant’s reference value (ECSC) or Peak Expiratory Flow (PEF) <80% of the participant’s individual optimal value measured at the screening visit,
14. Previous or ongoing respiratory tract infection (e.g., flu, COVID-19) and/or exacerbation of asthma within 4 weeks before the screening visit,
15. History of significant renal disease or chronic hepatic disease,
16. Malignant active disease (ongoing or within the five past years),
17. Severe autoimmune disease,
18. Immune defects including immunosuppression, immunopathies,
19. Vaccination during the entire treatment period, except flu and SARS-CoV-2 vaccinations,
20. Use of systemic immunosuppressive medications (e.g., methotrexate or cyclosporine A) or blood transfusion from one month before screening until the end of the trial,
21. General inflammatory, severe acute or chronic inflammatory diseases,
22. Other chronic diseases such as severe congestive heart failure, cardiovascular insufficiency, active gastric ulcer, inflammatory bowel disease, uncontrolled diabetes mellitus, etc.
23. Intake of antidepressant drugs with potent antihistamine properties such as tricyclic antidepressants (e.g., doxepin, amitriptyline, desipramine, imipramine, etc.),
24. Administration or planned administration of anti-IgE antibodies, mast cell stabilizers or anti-leukotriene agents,
25. Intake of beta-blockers/ACE inhibitor medication (angiotensin-converting enzyme inhibitor),
26. Active tuberculosis,
27. Having any contraindication for the use of adrenaline (including hyperthyroidism),
28. Known positive serology to Human Immunodeficiency Virus-1/2, Hepatitis B Virus or Hepatitis C Virus,
29. Females who were pregnant, lactating, or of child-bearing potential and not using a highly effective contraceptive method,
30. Administration of corticosteroids (systemic or nasal) or of anti-histaminic drugs within a defined time period preceding the trial (screening visit), as defined in the section Screening/Baseline Assessments and Procedures; exception made for routine (previously prescribed) control medication for asthmatic participants,
31. Laboratory values
    a. Missing laboratory values relevant for inclusion.
    b. Participants with a clinically significant sensitization to other environmental allergens (i.e., house dust mites, cat dander, dog dander) and whose CAP class of the respective allergen specific IgE was higher than for birch pollen specific IgE (t3),
    c. Clinically relevant laboratory values (haematology, clinical chemistry), grade ≥2 according to the FDA Guidance for Industry for preventive Vaccine Trials (FDA 2007) at screening visit (Participants with laboratory values ≥ grade 2 required retesting. Upon normalization of the out-of-range value(s), participant was eligible).

# Supplementary Material S2. Treatment schedule

| **Visit**  **No.** | **Volume of injection** | **Group I**  **0 mTU/mL (Placebo)** | **Group II**  **10 000 mTU/mL** |
| --- | --- | --- | --- |
| **T1** | **0.1 mL** | **0 mTU** | **1000 mTU** |
|  | **0.2 mL** | **0 mTU** | **2000 mTU** |
| **T2** | **0.2 mL** | **0 mTU** | **2000 mTU** |
|  | **0.3 mL** | **0 mTU** | **3000 mTU** |
| **T3** | **0.5 mL** | **0 mTU** | **5000 mTU** |
| **T4** | **0.5 mL** | **0 mTU** | **5000 mTU** |
| **T5** | **0.5 mL** | **0 mTU** | **5000 mTU** |
| **T6** | **0.5 mL** | **0 mTU** | **5000 mTU** |
| **Total cumulative dose** | | **0 mTU** | **28 000 mTU** |

# Supplementary Material S3. Trial Sites distribution with regard to the pollen stations available via the German Pollen Information Service as well as start/stop dates of the peak/entire pollen season and duration according to EAACI (Pfaar et al., ^10^)

| **Pollen station** | **Location** | **(EPS) start** | **Surrogate EPS start** | **PPS start** | **Surrogate PPS start** | **Surrogate PPS end** | **PPS end** | **Surrogate EPS end** | **EPS end** | **days EPS** | **days surrogate EPS** | **days PPS** | **days surrogate PPS** |
| --- | --- | --- | --- | --- | --- | --- | --- | --- | --- | --- | --- | --- | --- |
| Berlin | Neuenhagen, Brandenburg | 27-MAR-2025 | 27-MAR-2025 | 03-APR-2025 | 03-APR-2025 | 18-APR-2025 | 18-APR-2025 | 25-APR-2025 | 05-MAY-2025 | 40 | 30 | 16 | 16 |
| Bochum | Wuppertal, Nordrhein-Westfalen | 24-MAR-2025 | 24-MAR-2025 | 02-APR-2025 | 02-APR-2025 | 13-APR-2025 | 13-APR-2025 | 21-APR-2025 | 21-APR-2025 | 29 | 29 | 12 | 12 |
| Bochum | Duisburg, Nordrhein-Westfalen | 24-MAR-2025 | 03-APR-2025 | 02-APR-2025 | 03-APR-2025 | 16-APR-2025 | 13-APR-2025 | 02-MAY-2025 | 21-APR-2025 | 29 | 30 | 12 | 14 |
| Bochum | Essen, Nordrhein-Westfalen | 24-MAR-2025 | 02-APR-2025 | 02-APR-2025 | 02-APR-2025 | 13-APR-2025 | 13-APR-2025 | 01-MAY-2025 | 21-APR-2025 | 29 | 30 | 12 | 12 |
| Bonn | Köln, Nordrhein-Westfalen | 25-MAR-2025 | 31-MAR-2025 | 27-MAR-2025 | 31-MAR-2025 | 13-APR-2025 | 09-APR-2025 | 29-APR-2025 | 15-APR-2025 | 22 | 30 | 14 | 14 |
| Bonn | Bonn, Nordrhein-Westfalen | 25-MAR-2025 | 31-MAR-2025 | 27-MAR-2025 | 31-MAR-2025 | 13-APR-2025 | 09-APR-2025 | 29-APR-2025 | 15-APR-2025 | 22 | 30 | 14 | 14 |
| Borstel | Itzehoe, Schleswig-Holstein | 01-APR-2025 | 01-APR-2025 | 12-APR-2025 | 12-APR-2025 | 22-APR-2025 | 22-APR-2025 | 08-MAY-2025 | 08-MAY-2025 | 38 | 38 | 11 | 11 |
| Borstel | Hamburg, Hamburg | 01-APR-2025 | 01-APR-2025 | 12-APR-2025 | 12-APR-2025 | 22-APR-2025 | 22-APR-2025 | 08-MAY-2025 | 08-MAY-2025 | 38 | 38 | 11 | 11 |
| Chemnitz | Leipzig, Sachsen | 01-APR-2025 | 01-APR-2025 | 09-APR-2025 | 09-APR-2025 | 22-APR-2025 | 22-APR-2025 | 28-APR-2025 | 28-APR-2025 | 28 | 28 | 14 | 14 |
| Chemnitz | Saalfeld/ Saale, Thueringen | 01-APR-2025 | 01-APR-2025 | 09-APR-2025 | 09-APR-2025 | 22-APR-2025 | 22-APR-2025 | 28-APR-2025 | 28-APR-2025 | 28 | 28 | 14 | 14 |
| Chemnitz | Dresden, Sachsen | 01-APR-2025 | 01-APR-2025 | 09-APR-2025 | 09-APR-2025 | 22-APR-2025 | 22-APR-2025 | 28-APR-2025 | 28-APR-2025 | 28 | 28 | 14 | 14 |
| Chemnitz | Dresden, Sachsen | 01-APR-2025 | 01-APR-2025 | 09-APR-2025 | 09-APR-2025 | 22-APR-2025 | 22-APR-2025 | 28-APR-2025 | 28-APR-2025 | 28 | 28 | 14 | 14 |
| Chemnitz | Leipzig, Sachsen | 01-APR-2025 | 01-APR-2025 | 09-APR-2025 | 09-APR-2025 | 22-APR-2025 | 22-APR-2025 | 28-APR-2025 | 28-APR-2025 | 28 | 28 | 14 | 14 |
| Chemnitz | Dresden, Sachsen | 01-APR-2025 | 01-APR-2025 | 09-APR-2025 | 09-APR-2025 | 22-APR-2025 | 22-APR-2025 | 28-APR-2025 | 28-APR-2025 | 28 | 28 | 14 | 14 |
| Delmenhorst | Peine, Niedersachsen | 28-MAR-2025 | 28-MAR-2025 | 02-APR-2025 | 02-APR-2025 | 16-APR-2025 | 16-APR-2025 | 03-MAY-2025 | 03-MAY-2025 | 37 | 37 | 15 | 15 |
| Löwenstein | Heidelberg, Baden-Wuerttemberg | 04-APR-2025 | 04-APR-2025 | 04-APR-2025 | 04-APR-2025 | 17-APR-2025 | 17-APR-2025 | 17-APR-2025 | 17-APR-2025 | 14 | 14 | 14 | 14 |
| Löwenstein | Schorndorf, Baden-Wuerttemberg | 04-APR-2025 | 04-APR-2025 | 04-APR-2025 | 04-APR-2025 | 17-APR-2025 | 17-APR-2025 | 17-APR-2025 | 17-APR-2025 | 14 | 14 | 14 | 14 |
| Marburg | Dreieich, Hessen | 01-APR-2025 | 01-APR-2025 | 04-APR-2025 | 04-APR-2025 | 17-APR-2025 | 17-APR-2025 | 20-APR-2025 | 20-APR-2025 | 20 | 20 | 14 | 14 |
| Marburg | Wiesbaden, Hessen | 01-APR-2025 | 01-APR-2025 | 04-APR-2025 | 04-APR-2025 | 17-APR-2025 | 17-APR-2025 | 20-APR-2025 | 20-APR-2025 | 20 | 20 | 14 | 14 |
| Marburg | Marburg, Hessen | 01-APR-2025 | 01-APR-2025 | 04-APR-2025 | 04-APR-2025 | 17-APR-2025 | 17-APR-2025 | 20-APR-2025 | 20-APR-2025 | 20 | 20 | 14 | 14 |
| Marburg | Frankfurt am Main, Hessen | 01-APR-2025 | 01-APR-2025 | 04-APR-2025 | 04-APR-2025 | 17-APR-2025 | 17-APR-2025 | 20-APR-2025 | 20-APR-2025 | 20 | 20 | 14 | 14 |
| München | München, Bayern | 03-APR-2025 | 03-APR-2025 | 09-APR-2025 | 09-APR-2025 | 22-APR-2025 | 22-APR-2025 | 25-APR-2025 | 25-APR-2025 | 23 | 23 | 14 | 14 |

# Supplementary Material S4. Baseline characteristics – all participants and subgroup analysis (adults and adolescents)

**All participants**

| **Characteristics** | **Placebo** | **EP-088_T502** | **Total** |
| --- | --- | --- | --- |
| Participants (n) (%) | 92 (33.1%) | 186 (66.9%) | 278 (100%) |
| Female (n) (%) | 50 (54.3%) | 91 (48.9%) | 141 (50.7%) |
| Male (n) (%) | 42 (45.7%) | 95 (51.1%) | 137 (49.3%) |
| Age mean ± SD (years) | 39 ± 13 | 36 ± 12 | 37 ± 13 |
| Height mean ± SD (cm) | 174 ± 10 | 173 ± 10 | 174 ± 10 |
| Body weight mean ± SD (kg) | 81.6 ± 19.8 | 79.9 ± 18.4 | 80.5 ± 18.9 |
| Asthma (n) (%) | 26 (28.3%) | 42 (22.6%) | 68 (24.5%) |
| Allergic Asthma (n) (%) | 24 (92.3%) | 35 (83.3%) | 59 (86.8%) |

## **Adults**

| **Characteristics** | **Placebo** | **EP-088_T502** | **Total** |
| --- | --- | --- | --- |
| Participants (n) (%) | 88 (33.2%) | 177 (66.8%) | 265 (100%) |
| Female (n) (%) | 47 (53.4%) | 90 (50.8%) | 137 (51.7%) |
| Male (n) (%) | 41 (46.6%) | 87 (49.2%) | 128 (48.3%) |
| Age mean ± SD (years) | 40 ± 12 | 37 ± 12 | 38 ± 12 |
| Height mean ± SD (cm) | 174 ± 9 | 174 ± 10 | 174 ± 10 |
| Body weight mean ± SD (kg) | 82.7 ± 19.5 | 80.9 ± 18.1 | 81.5 ± 18.6 |
| Asthma (n) (%) | 25 (28.4%) | 41 (23.2%) | 66 (24.9%) |
| Allergic Asthma (n) (%) | 23 (92%) | 35 (85.4%) | 58 (87.9%) |

## **Adolescents**

| **Characteristics** | **Placebo** | **EP-088_T502** | **Total** |
| --- | --- | --- | --- |
| Participants (n) (%) | 4 (30.8%) | 9 (69.2%) | 13 (100%) |
| Female (n) (%) | 3 (75%) | 1 (11.1%) | 4 (30.8%) |
| Male (n) (%) | 1 (25%) | 8 (88.9%) | 9 (69.2%) |
| Age mean ± SD (years) | 14 ± 1 | 14 ± 2 | 14 ± 2 |
| Height mean ± SD (cm) | 167 ± 12 | 167 ± 6 | 167 ± 8 |
| Body weight mean ± SD (kg) | 57.1 ± 10.2 | 60.6 ± 11.5 | 59.5 ± 10.8 |
| Asthma (n) (%) | 1 (25%) | 1 (11.1%) | 2 (15.4%) |
| Allergic Asthma (n) (%) | 1 (100%) | 0 (0%) | 1 (50%) |

# Supplementary Material S5. ARIA Questionnaire assessed at the screening visit.

|  | | | | **Placebo** | **Active** | | **Total** |
| --- | --- | --- | --- | --- | --- | --- | --- |
| **Are the rhinitis symptoms troublesome?** | no | N (%) | 0 (0%) | | 0 (0%) | 0 (0%) | |
|  | yes | N (%) | 92 (100%) | | 186 (100%) | 278 (100%) | |
|  | Total | N (%) | 92 (100%) | | 186 (100%) | 278 (100%) | |
| **Do the rhinitis symptoms cause impairment of the participant's daily activities, in sports, at leisure?** | no | N (%) | 0 (0%) | | 4 (2.2%) | 4 (1.4%) | |
|  | yes | N (%) | 92 (100%) | | 182 (97.8%) | 274 (98.6%) | |
|  | Total | N (%) | 92 (100%) | | 186 (100%) | 278 (100%) | |
| **Do the rhinitis symptoms cause impairment at work or at school?** | no | N (%) | 4 (4.3%) | | 11 (5.9%) | 15 (5.4%) | |
|  | yes | N (%) | 88 (95.7%) | | 175 (94.1%) | 263 (94.6%) | |
|  | Total | N (%) | 92 (100%) | | 186 (100%) | 278 (100%) | |
| **Do the rhinitis symptoms disturb the participant's sleep?** | no | N (%) | 13 (14.1%) | | 14 (7.5%) | 27 (9.7%) | |
|  | yes | N (%) | 79 (85.9%) | | 172 (92.5%) | 251 (90.3%) | |
|  | Total | N (%) | 92 (100%) | | 186 (100%) | 278 (100%) | |
| **The allergic rhinitis is...** | Intermittent (symptoms are present < 4 days a week or for < 4 consecutive weeks) | N (%) | 12 (13%) | | 24 (12.9%) | 36 (12.9%) | |
|  | Persistent (symptoms are present > 4 days a week and for > 4 consecutive weeks) | N (%) | 80 (87%) | | 162 (87.1%) | 242 (87.1%) | |
|  | Total | N (%) | 92 (100%) | | 186 (100%) | 278 (100%) | |

# Supplementary Material S6. Frequency distribution of CAP classes for common silver birch sIgE (t3) at the screening visit.

| **CAP class** | **Placebo** | | **Active** | | **Total** | |
| --- | --- | --- | --- | --- | --- | --- |
|  | **Count** | **Valid N %** | **Count** | **Valid N %** | **Count** | **Valid N %** |
| 1 | 1 | 1.1% | 1 | 0.5% | 2 | 0.7% |
| 2 | 1 | 1.1% | 3 | 1.6% | 4 | 1.4% |
| 3 | 48 | 52.2% | 77 | 41.4% | 125 | 45.0% |
| 4 | 25 | 27.2% | 75 | 40.3% | 100 | 36.0% |
| 5 | 14 | 15.2% | 16 | 8.6% | 30 | 10.8% |
| 6 | 3 | 3.3% | 14 | 7.5% | 17 | 6.1% |
| Total | 92 | 100% | 186 | 100% | 278 | 100% |

**Supplementary Material S7.** Mean/median relative/absolute differences and effect size of the CSMS, dMS and dSS in patients treated with EP-088_T502 in comparison to placebo. As the dSS is identical in both scores, it is only displayed in table S7A.

A) Additive CSMS according to Mösges et al.^8^, B) CSMS according to EAACI (Pfaar et al.^11^)

FAS; Full Analysis Set, EPS: Entire Pollen Season, PPS: Peak Pollen Season, PP: Per Protocol Set.

**A)**

| **Population** | **Season** | **Score** | **Mean abs. diff. (Active − Placebo)** | **Median abs. diff.** | **Mean rel. diff. (%)** | **Median rel. diff. (%)** | **Effect size (mean / median)** |
| --- | --- | --- | --- | --- | --- | --- | --- |
| FAS | EPS | dSS | −0.10 | −0.14 | −11.80 | −17.56 | 0.20 / 0.28 |
| FAS | EPS | dMS | -0.20 | -0.13 | -26.15 | -22.06 | 0.32 / 0.21 |
| FAS | EPS | **CSMS** | -0.30 | -0.33 | -19.07 | -21.77 | 0.32 / 0.35 |
| FAS | PPS | dSS | −0.11 | −0.17 | −13.11 | −20.14 | 0.22 / 0.32 |
| FAS | PPS | dMS | -0.19 | -0.14 | -23.24 | -22.42 | 0.27 / 0.20 |
| FAS | PPS | **CSMS** | -0.31 | -0.26 | -18.404 | -17.37 | 0.30 / 0.25 |
| PP | EPS | dSS | -0.15 | -0.21 | -18.58 | -26.77 | 0.32 / 0.44 |
| PP | EPS | dMS | -0.21 | -0.16 | -27.12 | -27.15 | 0.32 / 0.25 |
| PP | EPS | **CSMS** | -0.36 | -0.41 | -22.74 | -28.31 | 0.38 / 0.43 |
| PP | PPS | dSS | -0.17 | -0.23 | -19.23 | -28.35 | 0.33 / 0.44 |
| PP | PPS | dMS | -0.19 | -0.21 | -24.13 | -34.12 | 0.27 / 0.29 |
| PP | PPS | **CSMS** | -0.36 | -0.33 | -21.61 | -22.80 | 0.35 / 0.32 |

**B)**

| **Population** | **Season** | **Score** | **Mean abs. diff. (Active − Placebo)** | **Median abs. diff.** | **Mean rel. diff. (%)** | **Median rel. diff. (%)** | **Effect size (mean / median)** |
| --- | --- | --- | --- | --- | --- | --- | --- |
| FAS | EPS | dMS | −0.18 | −0.15 | −35.1 | −32.6 | 0.35 / 0.90 |
| FAS | EPS | **CSMS** | −0.26 | −0.35 | −21.6 | −31.0 | 0.33 / 0.49 |
| FAS | PPS | dMS | −0.16 | −0.17 | −30.1 | −36.2 | 0.30 / 1.25 |
| FAS | PPS | **CSMS** | −0.27 | −0.32 | −21.2 | −26.3 | 0.31 / 0.45 |
| PP | EPS | dMS | −0.17 | −0.16 | −34.8 | −41.2 | 0.33 / 1.09 |
| PP | EPS | **CSMS** | −0.31 | −0.45 | −26.7 | −45.5 | 0.38 / 0.69 |
| PP | PPS | dMS | −0.16 | −0.26 | −29.4 | −68.2 | 0.28 / 1.85 |
| PP | PPS | **CSMS** | −0.32 | −0.44 | −25.8 | −41.3 | 0.36 / 0.67 |

**Supplementary Material S8.** CSMS during the peak birch pollen season (FAS) - Sensitivity Analysis with DWD-Pollen data

Imputation as defined in SAP: MAR for missing values, if at least 50% are missing

|  | | | | | | | | | | | | | **Treatment Difference** | |  |
| --- | --- | --- | --- | --- | --- | --- | --- | --- | --- | --- | --- | --- | --- | --- | --- |
| **Variable** | **Treatment** | **n** | | **Mean** | **SD** | **SEM** | **Median** | | **Range** | | **25%-75% Percentiles** | **95% CI of Means** | **Estimate (pooled SD)** | **(95% CI)** | **p-Value** |
| CSMS | Active | 186 | | 1.357 | 0.978 | 0.07 | 1.200 | | 0.00-5.19 | | 0.63-1.85 | 1.22-1.50 | -.34 (1.02) | (-.60, -.09) | 0.0078 |
|  | Placebo | 92 | | 1.701 | 1.087 | 0.11 | 1.529 | | 0.00-4.98 | | 0.92-2.24 | 1.48-1.93 |  |  |  |
|  | | | ANOVA Sum of squares | df | Mean Square | F | p-Value |  | |  | |  |  |  |  |
| Corrected Model | | | 27.605 | 11 | 2.510 | 2.50 | 0.0053 |  | |  | |  |  |  |  |
| Treatment group | | | 7.230 | 1 | 7.230 | 7.20 | 0.0078 |  | |  | |  |  |  |  |
| Pooled trial region according to DWD | | | 20.375 | 10 | 2.038 | 2.03 | 0.0308 |  | |  | |  |  |  |  |
| _______________________________________________________________________________ Active: EP-088_T502, SD: Standard Deviation, SEM: Standard Error of Mean, CI: Confidence Interval Note: p-value from ANOVA with treatment group as a fixed effect and pooled trial site as a covariate Note: Trial sites are pooled across the same pollen-measuring station T_CSMS_T.sas 10OCT25 06:58 | | | | | | | | | | | | | | | |

## Sensitivity analysis

| **CSMS during the peak birch pollen season (FAS) – ANOVA   with pooled trial site as covariate PID data** | **Treatment** | **Valid N** | **Mean (SD)** | **Pooled SD** | **Absolute difference  (Placebo-Active)** | **Relative difference  (Placebo-Active)/Placebo)** | **Ratio (absolute difference/pooled SD)** |
| --- | --- | --- | --- | --- | --- | --- | --- |
|  | **Active** | 186 | 1.37(0.98) | 1.01 | 0.30 | 18% | 29.7% |
|  | **Placebo** | 92 | 1.67(1.05) |  |  |  |  |
| **CSMS during the peak birch pollen season (FAS) – sensitivity analysis with DWD-Pollen data** | **Treatment** | **n** | **Mean (SD)** | **Pooled SD** | **Absolute difference  (Placebo-Active)** | **Relative difference  (Placebo-Active)/Placebo)** | **Ratio (absolute difference/pooled SD)** |
|  | **Active** | 186 | 1.36(0.98) | 1.02 | 0.34 | 20% | 33.3% |
|  | **Placebo** | 92 | 1.70(1.09) |  |  |  |  |

**Supplementary Material S9**. Bet v 1-specific IgE levels before and after treatment with EP-088_T502 or placebo. Grey bars indicate placebo, and blue bars indicate EP-088_T502. Data are presented as mean ± SD. ***p<0.0001; n.s., not statistically significant.

## **Supplementary Material S10.** Frequency of injection site reactions in all participants (SAF)

**All participants**

|  | **Placebo (N=84)** | | **Active (N=495)** | | **Total (N=579)** | |
| --- | --- | --- | --- | --- | --- | --- |
| Injection site discomfort | 0 | 0% | 1 | 0.20% | 1 | 0.17% |
| Injection site erythema | 0 | 0% | 2 | 0.40% | 2 | 0.35% |
| Injection site pain | 1 | 1.19% | 16 | 3.23% | 17 | 2.94% |
| Injection site pruritus | 2 | 2.38% | 162 | 32.73% | 164 | 28.32% |
| Injection site reaction | 1 | 1.19% | 15 | 3.03% | 16 | 2.76% |
| Injection site swelling | 1 | 1.19% | 73 | 14.75% | 74 | 12.78% |
| Injection site warmth | 1 | 1.19% | 34 | 6.87% | 35 | 6.04% |
| Local reaction | 0 | 0% | 4 | 0.81% | 4 | 0.69% |
| Total | 6 | 7.14% | 307 | 62.02% | 313 | 54.06% |

## **Subgroup analysis in adolescents and adults**

|  | **Placebo** | | **EP-088_T502** | | | |
| --- | --- | --- | --- | --- | --- | --- |
|  | **Adults (N=84)** | | **Adolescents (N=17)** | | **Adults (N=478)** | |
| Injection site discomfort | 0 | 0% | 0 | 0% | 1 | 0.21% |
| Injection site erythema | 0 | 0% | 0 | 0% | 2 | 0.42% |
| Injection site pain | 1 | 1.19% | 0 | 0% | 16 | 3.35% |
| Injection site pruritus | 2 | 2.38% | 2 | 11.76% | 160 | 33.47% |
| Injection site reaction | 1 | 1.19% | 2 | 11.76% | 13 | 2.72% |
| Injection site swelling | 1 | 1.19% | 6 | 35.29% | 67 | 14.02% |
| Injection site warmth | 1 | 1.19% | 1 | 5.88% | 33 | 6.90% |
| Local reaction | 0 | 0% | 0 | 0% | 4 | 0.84% |
| Total | 6 | 7.14% | 11 | 64.71% | 296 | 61.92% |

**Supplementary Material S11.** Systemic reaction classified as systemic adverse drug reactions

| **Treatment group** | **Description of systemic reaction** | **Severity** | **Outcome** | **Action taken with IMP** |
| --- | --- | --- | --- | --- |
| EP-088_T502 | Late phase SR Grade III after T1:  Systemic allergic reaction (exanthema, dyspnea, eye angioedema, wheezing and localised itching) | Severe | Resolved within 6 hours after initial treatment at the trial site (crystalloid solution, prednisolone, clemastine, and salbutamol) and subsequent hospitalisation | Discontinued from treatment |

**Supplementary Material S12.** Frequency of local reactions (LRs) – wheal diameters at the injection site

## Immediate LRs in the EP-088_T502 and placebo treatment groups by category

| **Wheal size [cm]** | **Adolescents** | | | | | | **Adults** | | | | | | **Total** | | | | | |
| --- | --- | --- | --- | --- | --- | --- | --- | --- | --- | --- | --- | --- | --- | --- | --- | --- | --- | --- |
|  | **Placebo** | | **EP-088_T502** | | **Total** | | **Placebo** | | **EP-088_T502** | | **Total** | | **Placebo** | | **EP-088_T502** | | **Total** | |
| **Valid** | **N** | **%** | **N** | **%** | **N** | **%** | **N** | **%** | **N** | **%** | **N** | **%** | **N** | **%** | **N** | **%** | **N** | **%** |
| **0 cm** | 32 | 100% | 35 | 48.6% | 67 | 64.4% | 554 | 80.3% | 467 | 34.6% | 1021 | 50% | 586 | 81.2% | 502 | 35.3% | 1088 | 50.7% |
| **<5 cm (mild)** | 0 | 0% | 37 | 51.4% | 37 | 35.6% | 136 | 19.7% | 879 | 65.1% | 1015 | 49.8% | 136 | 18.8% | 916 | 64.4% | 1052 | 49.1% |
| **5-10 cm (moderate)** | 0 | 0% | 0 | 0% | 0 | 0% | 0 | 0% | 4 | 0.3% | 4 | 0.2% | 0 | 0% | 4 | 0.3% | 4 | 0.2% |
| **>10 cm (severe)** | 0 | 0% | 0 | 0% | 0 | 0% | 0 | 0% | 0 | 0% | 0 | 0% | 0 | 0% | 0 | 0% | 0 | 0% |
| **Total** | 32 | 100% | 72 | 100% | 104 | 100% | 690 | 100% | 1350 | 100% | 2040 | 100% | 722 | 100% | 1422 | 100% | 2144 | 100% |

## Late phase LRs in the EP-088_T502 and placebo treatment groups by category

| **Wheal size [cm]** | **Adolescents** | | | | | | **Adults** | | | | | | **Total** | | | | | |
| --- | --- | --- | --- | --- | --- | --- | --- | --- | --- | --- | --- | --- | --- | --- | --- | --- | --- | --- |
|  | **Placebo** | | **EP-088_T502** | | **Total** | | **Placebo** | | **EP-088_T502** | | **Total** | | **Placebo** | | **EP-088_T502** | | **Total** | |
| **Valid** | **N** | **%** | **N** | **%** | **N** | **%** | **N** | **%** | **N** | **%** | **N** | **%** | **N** | **%** | **N** | **%** | **N** | **%** |
| **0 cm** | **75** | 98.7% | 118 | 65.2% | 193 | 75.1% | 1682 | 95.6% | 2496 | 69.8% | 4178 | 78.3% | 1757 | 95.7% | 2614 | 69.5% | 4371 | 78.1% |
| **<10 cm (mild)** | **1** | 1.3% | 62 | 34.3% | 63 | 24.5% | 78 | 4.4% | 1019 | 28.5% | 1097 | 20.6% | 79 | 4.3% | 1081 | 28.8% | 1160 | 20.7% |
| **10-20 cm (moderate)** | **0** | 0% | 0 | 0% | 0 | 0% | 0 | 0% | 62 | 1.7% | 62 | 1.2% | 0 | 0% | 62 | 1.6% | 62 | 1.1% |
| **>20 cm (severe)** | **0** | 0% | 1 | 0.6% | 1 | 0.4% | 0 | 0% | 1 | 0% | 1 | 0% | 0 | 0% | 2 | 0.1% | 2 | 0% |
| **Total** | **76** | 100% | 181 | 100% | 257 | 100% | 1760 | 100% | 3578 | 100% | 5338 | 100% | 1836 | 100% | 3759 | 100% | 5595 | 100% |

**Supplementary Material S13.** AdolRQLQ scores in adolescents before treatment (T1), before the birch pollen season (O1), and during the birch pollen season (O2). Data are presented as mean ± SD. n.s., not statistically significant.
